# Task-evoked Negative BOLD Response and Functional Connectivity in the Default Mode Network are Representative of Two Overlapping but Separate Neurophysiological Processes

**DOI:** 10.1038/s41598-019-50483-8

**Published:** 2019-10-09

**Authors:** David B. Parker, Qolamreza R. Razlighi

**Affiliations:** 1 Department of Biomedical Engineering, Columbia University, New York, NY 10027 USA; 2 Department of Neurology, College of Physicians and Surgeons, Columbia University Medial Center, New York, NY 10032 USA; 3 Taub Institute for research on Alzheimer’s disease and the aging brain, Columbia University Medical Center, New York, NY 10032 USA

**Keywords:** Attention, Diagnostic markers

## Abstract

The topography of the default mode network (DMN) can be obtained with one of two different functional magnetic resonance imaging (fMRI) methods: either from the spontaneous but organized synchrony of the low-frequency fluctuations in resting-state fMRI (rs-fMRI), known as “*functional connectivity*”, or from the consistent and robust deactivations in task-based fMRI (tb-fMRI), here referred to as the “*negative BOLD response*” (NBR). These two methods are fundamentally different, but their results are often used interchangeably to describe the brain’s resting-state, baseline, or intrinsic activity. While the DMN was initially defined by consistent task-based decreases in blood flow in a set of specific brain regions using PET imaging, recently nearly all studies on the DMN employ functional connectivity in rs-fMRI. In this study, we first show the high level of spatial overlap between NBR and functional connectivity of the DMN extracted from the same tb-fMRI scan; then, we demonstrate that the NBR in putative DMN regions can be significantly altered without causing any change in their overlapping functional connectivity. Furthermore, we present evidence that in the DMN, the NBR is more closely related to task performance than the functional connectivity. We conclude that the NBR and functional connectivity of the DMN reflect two separate but overlapping neurophysiological processes, and thus should be differentiated in studies investigating brain-behavior relationships in both healthy and diseased populations. Our findings further raise the possibility that the macro-scale networks of the human brain might internally exhibit a hierarchical functional architecture.

## Introduction

The default mode network, or DMN, is a set of functionally interconnected brain regions whose activity consistently decreases during goal-oriented external tasks and/or increases during wakeful rest^1,2^. This pattern of activity has led to the hypothesis that the DMN plays a key role in orchestrating cognitive functions such as introspection, prospective memory, and a variety of processes described intuitively as daydreaming or mind-wandering^3,4^. Dysfunction of the DMN has also been implicated in pathological states including Alzheimer’s disease^5^, Parkinson’s disease^6^, autism^7^, schizophrenia^8^, and depression^9^.

Nowadays, the most common imaging technique used to investigate the DMN is functional magnetic resonance imaging (fMRI). The fMRI signal reflects the change in hemodynamics within a macro-scale (1~27 mm^3^) brain region, which is related to that region’s neural activity through a cascade of events referred to as *neurovascular coupling*^10–12^. Henceforth, any coherent activation or suppression (either spontaneously or in response to external stimuli) of a population of neurons and/or glial cells that is spatially localized in a macro-scale brain region and gives rise to a change in the fMRI signal will be referred to as the *neurophysiological process*.

Best practices for measuring and interpreting the fMRI signal have evolved over time and remain a topic of debate. For instance, the task-based fMRI (tb-fMRI) experiments were initially proposed to study task-responsive neurophysiological processes during presentation of various cognitive tasks or stimuli^13^. This paradigm was accompanied by the introduction of the blood oxygenation level dependent (BOLD) signal, although it is important to appreciate that the extracted BOLD signal only accounts for a small portion of the total fMRI signal variance^14^. More recently, ongoing or spontaneous neural activity has been hypothesized to induce inter-regional, coherent, and low-frequency fluctuations in the fMRI signal. These coherent fluctuations are believed to reflect functionally connected neurophysiological processes taking place within separate and distal macro-scale brain regions^15–17^. Therefore, it follows that the fMRI signal from a macro-scale brain region could simultaneously reflect both task-evoked and spontaneous neurophysiological processes. In fact, it has been reported that in any activated region, the fMRI signal is a combination of task-evoked and spontaneous neural activity^18^; however, other studies investigating the relationship between task-evoked and spontaneous activity in the brain suggest a more complex^19–22^, non-linear^23,24^, and even causal relationship^25–27^ between the two types of activity.

In this paper, our focus is on the DMN. The neurophysiological processes localized to DMN regions have been shown to be strongly task-responsive^28^ as well as functionally connected^16,29^. These two distinct characteristics of the DMN are often overlooked and considered to be a result of using two different techniques for investigating the same neurophysiological process^30,31^. One method, consistent with its initial identification^1,2^, defines the DMN based on task-driven deactivation, here referred to as task-evoked negative BOLD response (NBR). Another method defines the DMN based on correlated low-frequency fluctuations in the fMRI signal even in the absence of cognitive tasks or stimuli. This approach, known as resting-state functional connectivity MRI, has become the method of choice for many studies of whole-brain networks, owing in part to its convenience and minimal task demands. The use of two very different approaches has contributed to conflicting results and interpretations regarding the role of the DMN in normal and diseased brain function^32–35^. Furthermore, intrinsically oriented but externally presented tasks such as tasks of introspection, autobiographical memory retrieval, future planning, and self-referential processing are shown to activate the DMN regions^36–38^. It is therefore crucial to elucidate the differences between these two types of fMRI measurements, and to establish the relationship between task-based networks of co-activated or co-deactivated regions and task-independent functional connectivity networks in the same regions.

In a series of seminal meta-analyses, Smith *et al*. and others^39–42^ showed a high degree of spatial overlap between task-based co-activation networks and resting-state functional connectivity networks, concluding that regions in task-based co-activation networks continue to work together even after termination of the task^43^. This conclusion assumes that both task-based networks and functional connectivity networks reflect the spatial organization of a common neurophysiological process, and that task performance may only alter the temporal characteristics of the process. However, a recent study of ours demonstrated that performing a task does not alter the time-course or spatial pattern of functional connectivity in DMN regions^44^. These findings provided preliminary evidence for the existence of two distinct but spatially overlapping neurophysiological processes in the DMN regions, both of which are operational during task performance but may have very different functional roles. Therefore, we hypothesize that —at least in the DMN— the neurophysiological processes underlying functional connectivity and the NBR are distinct, and thus can be dissociated, and that the NBR reflects neurophysiological processes that are more closely involved in task performance than the functional connectivity. Our hypothesis, if it is true, would support the possibility of a hierarchical functional architecture in the macro-scale networks of the brain, in which spontaneous activity provides the scaffolding or infrastructure for neural processes that are involved in actual task execution.

To test whether and how the functional connectivity of the DMN and its task-based NBR can be dissociated, we used attention as a modulating factor to alter NBR in the DMN regions. This manipulation had no significant effect on the underlying functional connectivity of the same regions from which the NBR was extracted. Using two sensory-motor tb-fMRI scans and one rs-fMRI scan, we first show that there is a significant degree of spatial overlap of the functional connectivity networks extracted from the three scans, as well as the spatial extent of the NBR extracted from the two tb-fMRI scans. We found that switching attention from one sensory modality to another modulated the NBR in DMN regions while their functional connectivity remained unchanged, suggesting a disassociation between the two simultaneous fMRI measurements from the same regions. To asses which signal is more closely related to the actual task execution, we examined the subject-wise task performance as a function of the strength of functional connectivity as well as the magnitude of the NBR. We found that the magnitude of the NBR in the DMN regions was correlated with task performance, whereas the strength of functional connectivity in the same regions did not correlate with task performance. This suggests that the NBR is a closer proxy for neurophysiological processes involved in execution of the task compared to the functional connectivity obtained simultaneously from the same regions. We also replicate the findings using the Human Connectome Project dataset^45^, an open access, online tb/rs-fMRI dataset, acquired during a working memory task. Our findings provide evidence for the existence of two spatially overlapping but dissociable neurophysiological processes in the DMN that are simultaneously active and manifest as two different fMRI measurements.

## Methods

We examined NBR and functional connectivity using two separate datasets with completely different pools of participants, scanners, and pulse sequences, which were collected in two different research centers. Our primary sample (Dataset I) and its associated task were specifically designed for this study, whereas the replication sample (Dataset II) was chosen from a publicly available, online database.

### Participants

Dataset I included 30 young, healthy, right-handed participants (age = 25 ± 3.5 years, m/f = 10/20) recruited from the Columbia University Medical Center. All participants were compensated and signed an informed consent form prior to participation in the study. All research procedures were performed in accordance with relevant guidelines and regulations as approved by the Columbia University Institutional Review Board.

Dataset II included 100 young, healthy participants (age range = 22–35 years, m/f = 52/48) referred to as *“Unrelated-100”* in the Human Connectome Project^45^. Written informed consent was obtained from each participant in accordance with relevant guidelines and regulations approved by the local Institutional Review Board at Washington University in St. Louis. This dataset is available online, and the details of recruitment and demographics can be found elsewhere^46,47^.

### fMRI experimental design

**Figure 1:**
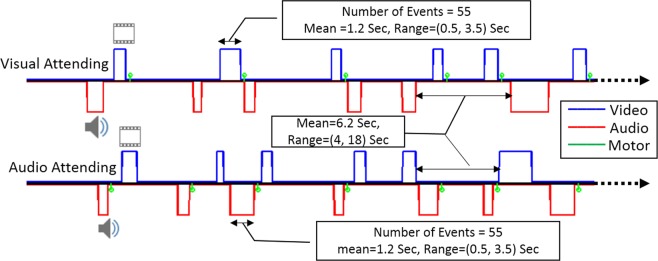
The design of the attention switching sensory-motor tasks using an event-related fMRI paradigm. The blue line denotes the random timing for duration and onset of the visual stimuli, and the red line shows the same for audio stimuli in a typical participant. The green bars represent the time of the response to either the visual or audio stimulus. There were at least 55 visual and 55 audio stimuli in each run, with mean duration of 1.2 sec and a range of 0.5 to 3.5 seconds. In one run, participants were instructed to attend to visual stimuli (top row) and press a button as soon as each visual stimulus was terminated, while ignoring audio stimuli. In another run, they were instructed to attend to audio stimuli (bottom row) and press a button as soon as each audio stimulus was terminated, while ignoring visual stimuli.

For Dataset I, we used an event-related fMRI paradigm with a visual-audio attention switching task. Subjects were presented with a sequence of visual and audio stimuli (flashing checkerboard and alternating tone) with random onsets and durations. The event duration was sampled from a uniform distribution (range: 0.4/0.6 ~ 4.0/4.0 s for visual/audio) and the onsets of the stimuli were jittered by applying a uniformly distributed inter-stimulus interval (range: 2.5 ~ 9.0 s). Two runs were collected for each participant; in one run, participants were asked to attend only to the visual stimuli and press a button with their right index finger at the end of each visual stimulus, ignoring the audio stimuli. In another run, participants were asked to attend only to the audio stimuli and press a button with their right index finger at the end of each audio stimulus, ignoring the visual stimuli. Figure 1 shows the design and timing of the fMRI paradigm that we used for this set of participants. Each fMRI run included at least 55 visual (blue) and 55 audio (red) stimuli which were allowed to overlap. The green bars illustrate the times when responses were made. The order of acquisition of the two runs (visual-attended and audio-attended) was randomized to prevent any systematic bias toward either stimulus modality. For half of the participants, a rs-fMRI scan with the same duration was collected as well.

For Dataset II, we used the N-back task, a working memory task in which participants were presented with a random sequence of pictures of four different types of items (faces, tools, places, and body parts) and instructed to answer whether or not the currently presented item was the same as the one presented N items earlier. This task was chosen from among the available tasks in the Connectome tb-fMRI dataset because it has the longest scan time, and can be modeled as an event-related fMRI experiment to capture the full dynamics of the positive/negative BOLD response. Details of this task can be found elsewhere^48,49^.

### MRI acquisition parameters

All three fMRI scans in Dataset I were acquired using a 3.0 Tesla *Achieva* Philips scanner with a T_2_*-weighted echo-planar imaging (EPI) sequence [TR/TE = 1000/20 ms; flip-angle = 72°; FOV = 240 × 240 mm; matrix-size = 80 × 80; voxel-size = 3.0 × 3.0 × 5.5 mm; 18 axial slices]. The duration of both tb-fMRI scans was 6 minutes (360 volumes), and the same total duration and parameters were used for the rs-fMRI scan to match for statistical power. An accompanying T_1_-weighted magnetization-prepared rapid gradient-echo (MPRAGE) structural image was collected [TR/TE = 6.5/3 ms; flip-angle = 8°; FOV = 25.6 × 25.6 cm; matrix-size = 256 × 256; voxel-size = 1.0 × 1.0 × 1.0 mm; 165 axial slices] for localization and spatial normalization of the functional data in each participant.

The two fMRI scans in Dataset II (2-back memory task and resting-state) were acquired on a customized Siemens 3.0 Tesla *Connectome Skyra* scanner using a multiband EPI sequence [TR/TE = 720/33.1 ms; flip-angle = 52°; FOV = 208 × 180 mm; matrix-size = 104 × 90; voxel-size = 2.0 × 2.0 × 2.0 mm; 72 axial slices; multiband factor = 8]. The acquisition parameters were identical for both the tb-fMRI and the rs-fMRI scans in order to provide maximal compatibility between task and resting-state data. The duration of each rs-fMRI scan was about 15 minutes (1200 volumes), whereas tb-fMRI was about 5 minutes (405 volumes). The first run of the rs-fMRI scan (out of four rs-fMRI run) was truncated to its first 405 volumes to match the duration of the tb-fMRI scan. Only the left-to-right phase encoding scan was used for both tb-fMRI and rs-fMRI data; however, since the minimally preprocessed volumetric data was used, the right-to-left phase encoding scans were used to perform gradient unwarping and distortion correction. Structural images were acquired using the 3D MPRAGE T_1_-weighted sequence [TR/TE = 2400/2.14 ms; flip-angle = 8°; FOV = 224 × 224 mm; matrix-size = 320 × 320; voxel-size = 0.7 × 0.7 × 0.7 mm; 256 sagittal slices].

### Analysis of fMRI data

All fMRI data from Dataset I were analyzed using the FSL (V5.0.7) software package. As in our recently published study^44^, realignment of the fMRI scans was performed by rigid-body registration of all the volumes to the middle one. Then, slice timing correction was performed by shifting each slice’s time-series to the instant when the middle slice was acquired. High-pass filtering was done with a non-linear Gaussian kernel with cutoff frequency of 0.01 Hz. Spatial smoothing was then performed with FWHM = 5 mm. Spatial normalization was performed by rigid-body registration of the first fMRI volume to its T_1_-weighted structural image, and then by non-linear registration of the structural image to the MNI template. Finally, intensity normalization was performed by global scaling of the data to have a median of 10^4^ and then to have a normalized intensity range between their 20^th^ and 80^th^ percentiles. First-level general linear model (GLM) analysis was performed by modeling the fMRI data with two regressors of interest, which were obtained by convolving the canonical HRF with the timing (zero-one boxcar function) of the visual and audio stimulations. The results were fed into a second-level analysis to generate group-level maps of the activations and deactivations using the mixed-effects modeling technique implemented in FSL. To compute the subject-wise magnitude of the NBR in DMN regions, we averaged the significant point estimates (β) of the first-level analysis in each subject that were located within established DMN regions (posterior cingulate, precuneus, medial prefrontal cortex, middle temporal gyrus and hippocampus). These regional masks were obtained from the Desikan-Killiany atlas^50^ in MNI space, and were only used to separate the NBR in the DMN from the other types of NBR often observed in the vicinity of the positive BOLD response (PBR) and also in ventricular space. The same mask was used throughout this paper to locate significant voxels within the DMN for computing the magnitude/expression of the networks.

We used independent component analysis (ICA) to extract the spatial extent and time-course of functional connectivity networks. As performed in our recent study^44^, the same preprocessed data that were fed into the first-level GLM analysis were also fed into a multivariate exploratory linear optimized decomposition into independent components (MELODIC) analysis with temporal concatenation of multiple subjects^51^. The number of ICs was estimated automatically using the Laplace approximation to the Bayesian evidence of the model order^51,52^. Resting-state data were preprocessed in exactly the same way as the tb-fMRI data to prevent any biases toward a given processing stream. In particular, we did not apply low-pass filtering of the rs-fMRI data in order to be consistent with the tb-fMRI pipeline. The DMN IC was selected based on observing significant connectivity among all three main nodes of the network; 1) posterior cingulate, 2) medial frontal gyrus, and 3) bilateral angular gyrus. We used dual regression implemented in FSL to obtain subject-wise expression (coherence and magnitude) of the extracted DMN IC, and statistical inference was applied on the subject-wise expression of the network to assess any significant change in the coherence and/or magnitude of the functional connectivity fluctuations^53^. Subject-wise expression of the functional connectivity in DMN regions was obtained by averaging the significant point estimates (β) given by the dual regression technique that fell within the aforementioned DMN regional masks. To assess any significant change in pair-wise inter-regional coherence, seed-based correlation coefficient analysis was used among the three main nodes of the DMN (posterior cingulate, medial-orbito-frontal, and right/left angular gyri). At each region the seed coordinates were obtained by computing the centroid of the three voxels with maximum correlation with the time-course of the DMN IC in the three scans. The preprocessed fMRI signals from all voxels within a 10 mm radius of each seed were averaged to obtain that region’s time-course. Subject-wise inter-regional coherence was measured by computing Pearson’s correlation coefficient between the time-course of the regions, and Student t-tests were used to assess any significant difference in the correlations obtained from different scans.

Both tb-fMRI and rs-fMRI scans in Dataset II were already preprocessed and publicly available. We used the minimally preprocessed volumetric fMRI data, which went through multiple processing steps, including gradient unwarping, motion correction, fieldmap-based EPI distortion correction, brain boundary-based registration to structural scan, non-linear registration to MNI, and grand mean intensity normalization. The details of this pipeline can be found in^54^. We only added the same smoothing and intensity normalization as we used for preprocessing of Dataset I. The preprocessed fMRI data were fed into the first-level GLM analysis with two regressors (0-back and 2-back). We used an event-related statistical analysis to find the BOLD response associated with the interval from the presentation of the stimulus until a response has been made. We combined all four types of stimuli (faces, tools, places, and body parts) into one regressor for each N-back. The parametric maps obtained from the first-level were fed into a second-level analysis to obtain the group-level map of activations and deactivations using mixed-effects in FSL. The ICA analyses on both tb-fMRI and rs-fMRI scans were performed exactly the same way as in Dataset I, as well as the computation of the magnitude of the NBR and the strength of functional connectivity in the DMN.

## Results

Figure 1 illustrates the design and timing of the stimuli in our fMRI experiment for the attention switching task in one randomly selected participant. As seen from the time of the button press responses (green), the participant responded to the visual stimuli in the visual-attended run (top row) and to the audio stimuli in the audio-attended run (bottom row). Participants responded correctly on 99.1%/99.2% of the visual/audio stimulus presentations, where a correct response is defined as a button press within 3 seconds of stimulus termination.

### Spatial overlap between functional connectivity of the DMN and its NBR

**Figure 2:**
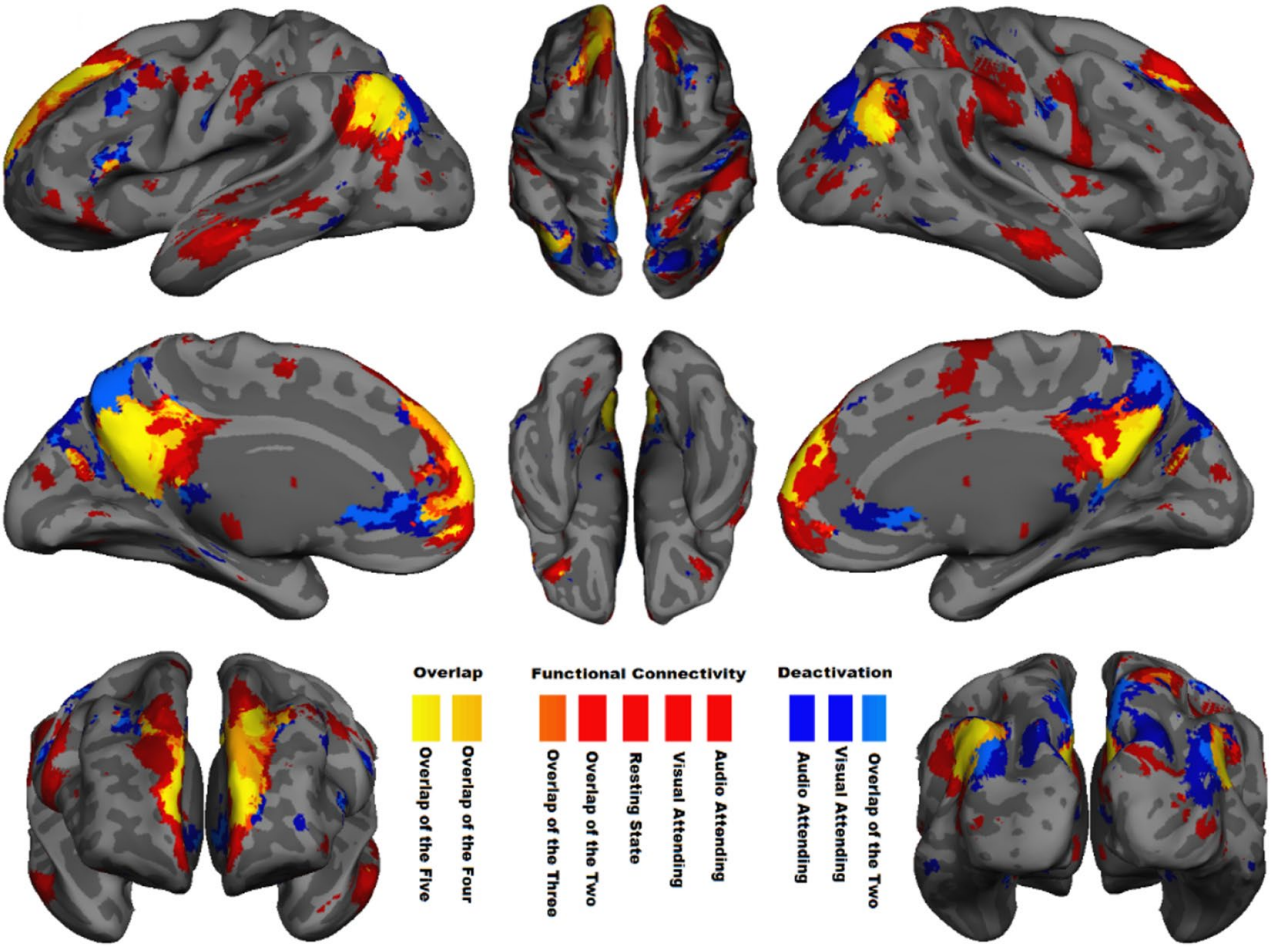
Spatial overlap between regions with significant negative BOLD response and functional connectivity in DMN. Voxel-wise significant z statistics are mapped onto a semi-inflated cortical surface for better visualization (slice-based mapping can be found in Fig. S1). Dark blue denotes the spatial extent of the regions with significant (z < −4 after multiple comparisons correction) NBR to visual- and audio-attended stimuli during two separate tb-fMRI scans. Light blue indicates their overlap. Red indicates the spatial extent of the regions with significant functional connectivity (z > 4 after multiple comparisons correction) extracted from two tb-fMRI scans (visual-attended and audio-attended tasks) as well as one rs-fMRI scan. Light red and orange highlight the overlap of the two and three functional connectivity networks, respectively. Dark-yellow and yellow indicate the overlap of four and all five of the aforementioned networks, respectively. See Fig. S2 for individually depicted and detailed spatial pattern of each functional connectivity network and task-related BOLD response illustrated in this figure.

Before testing whether functional connectivity strength can be dissociated from the NBR, we needed to assess the degree to which these signals arise from the same anatomical regions. First, we compared functional connectivity maps extracted from the two tb-fMRI scans with those extracted from the rs-fMRI scan, to ensure that the spatial pattern of DMN functional connectivity is similar during task performance and at rest. We ran separate group ICA (temporal concatenation) for each of the tb-fMRI runs, which resulted in 67/62 ICs where the DMN was the first component, accounting for 2.5%/3.6% of the variance for visual/audio-attended scan. We also ran group-level ICA for the available resting-state fMRI (15 participants) in which the DMN was detected as the sixth component out of 74 ICs, accounting for 1.7% of the total variance. Figure 2 shows the spatial pattern of these three DMN functional connectivity networks (|z| > 4 after cluster-wise multiple comparisons correction) overlaid on top of one another (in red) over a semi-inflated cortical surface (Fig. S1 shows the same on 36 axial slices, and Fig. S2 shows each spatial pattern individually). The pairwise spatial similarities of these functional connectivity networks are provided in Table 1 using the Dice similarity coefficient (DSC), indicating a significantly high spatial similarity between the extracted tb-fMRI and rs-fMRI networks (DSC = 0.61 and 0.50 for visual and auditory, respectively). Note that spatial similarity as low as DSC = 0.2 is extremely significant^40^ with p < 10^−5^, thus all the ICs with spatial similarity more than 0.2 have been illustrated in the supplementary Figs S4, S5 and S6 for the three scans. As seen in these figures, while there are some spatial similarities in parts of the posterior cingulate and angular gyrus, none of them show any connectivity in the medial frontal and bilateral middle temporal regions. In addition, they included regions that are not considered to be part of the DMN in the literature, thus we did not consider them in this study. Nonetheless, the high spatial similarities between the selected DMN ICs (Fig. 2) shows that the regions of the DMN are functionally connected irrespective of whether the participants are engaged in a task or are at rest, and they have a very similar spatial pattern of activity.

**Table 1:** Quantification of the pairwise spatial overlap of the regions with significant NBR and functional connectivity in the DMN using Dice similarity coefficient. These regions are depicted in Fig. 2.

		Functional connectivity of DMN			NBR of DMN regions	
		Attending Visual	Attending Audio	Resting State	Attending Visual	Attending Audio
Functional connectivity of DMN	Attending Visual		0.597	0.606	0.407	0.380
	Attending Audio	0.597		0.495	0.396	0.373
	Resting State	0.606	0.495		0.420	0.384
NBR of DMN	Attending Visual	0.407	0.396	0.421		0.567
	Attending Audio	0.380	0.374	0.384	0.568	

Second, we compared the NBR in DMN regions (extracted separately from two tb-fMRI scans) with the spatial pattern of functional connectivity extracted not only from the same tb-fMRI scans, but also from the separate rs-fMRI scan. Figure 2 shows the results of a second-level GLM analysis for attended visual and audio stimuli (|z| > 4 after cluster-wise multiple comparisons correction) in the DMN regions (dark blue and light blue). The overlap of these five networks —NBR in visual and audio tasks, and functional connectivity in the same tasks and at rest— has been color-coded from orange to yellow in Fig. 2 to illustrate the amount of spatial similarity among them. The pairwise spatial similarity between NBR and functional connectivity networks was highly significant (DSC = 0.37–0.57, p < 10^−8^ for each pairwise comparison; see Table 1), indicating a high degree of anatomical overlap of the neurophysiological processes that gives rise to these two signals in the DMN. However, there are also notable yet interesting differences in the spatial similarity of the NBR and functional connectivity. In particular, the NBRs extent towards the posteromedial cortex and more posteriorly in the frontal cortex. Conversely, the functional connectivity pattern extends from the posterior cingulate cortex towards the retrosplenial cortex. This is an interesting observation since the retrosplenial cortex is traditionally linked to introspection^55^, while the posteromedial cortex has at least one subpart related to externally-oriented cognitive tasks^56^, hinting at their distinct functionalities within the DMN regions.

### Attention modulates the NBR, but not functional connectivity, in the DMN

**Figure 3:**
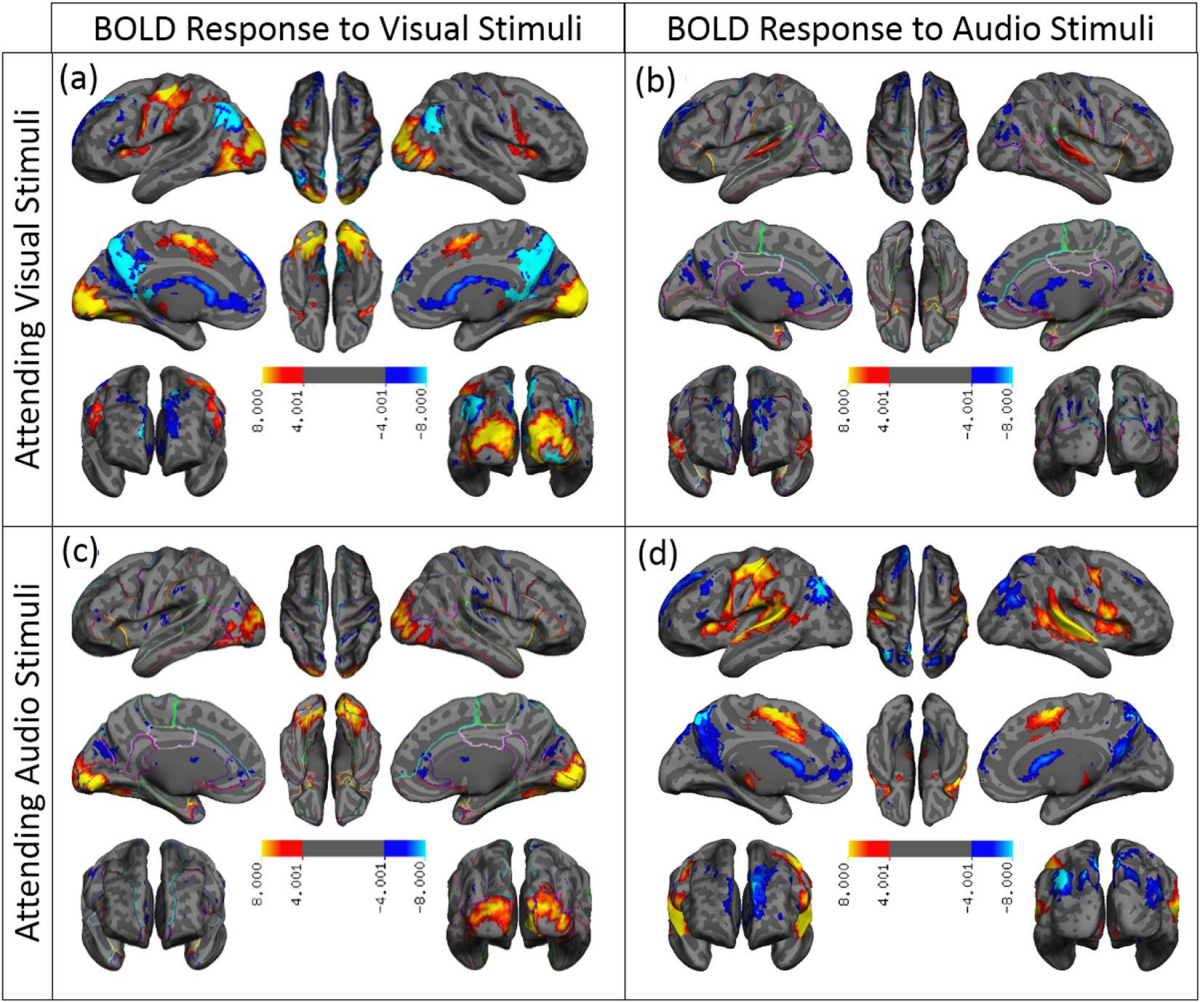
Attention-specificity of the negative BOLD response in the DMN regions. Spatial extent of the voxels with significant PBR (activated) and NBR (deactivated) during sensory-motor tasks using z-statistics thresholded at |z| > 4 for (**a**) attended visual, (**b**) unattended audio, (**c**) unattended visual, and (**d**) attended audio stimuli. The z-statistics for PBR are color-coded with warm colors (red-yellow), and those for NBR with cold colors (blue-light blue). The solid, light blue color represents the mask of the regions that have significantly higher magnitude of the NBR for attended stimuli versus unattended stimuli (z > 2.3, after cluster-wise multiple comparisons correction). The voxel-wise significant z statistics are mapped onto a semi-inflated cortical surface for a better visualization (slice-based mapping can be found in Fig. S3). Note that almost no significant NBR (deactivation) is present for unattended stimuli. The solid light blue color masks out most of the color-coded spatial maps of the deactivated area in the visual-attended BOLD response and partially in the audio-attended BOLD response. See the bottom row of the Fig. S2 for color-coded spatial maps of deactivated regions for both visual- and audio-attended tasks.

If the NBR and functional connectivity in the DMN reflect a common underlying neurophysiological process, then both signals should be similarly modulated by a demanding task manipulation. Conversely, a task-based modulation of NBR in the absence of a change in functionally connectivity strength would suggest distinct processes. To test this, we designed an experiment where we were able to significantly modulate the NBR in the DMN by shifting attention from one sensory modality to another and showed that such a shift in attention did not have any significant effect on the ongoing functional connectivity. In other words, we used the attention specificity of the NBR in the DMN to demonstrate the disassociation between NBR and functional connectivity in the DMN regions. Figure 3 shows the group-level activation map using z statistics (|z| > 4 after cluster-wise multiple comparisons correction) for visual (3a and 3c) and audio (3b and 3d) stimuli when the subjects were attending (3a and 3d) and when they were not attending (3b and 3c) to the stimuli. These activation maps are overlaid on a semi-inflated surface of the cerebral cortex and on 36 axial slices of the brain (Figs S3a to S3d in the same order). As expected, visual stimuli generated a PBR (illustrated by hot colors) in the primary visual cortex, while audio stimuli induced a PBR in the primary auditory cortex. Figure 3 also shows that when a stimulus, whether visual or audio, was the focus of attention, it generated a significant NBR (illustrated by cold colors) in the DMN regions (Fig. 3a,d), whereas when the same stimulus was ignored, it produced only scattered NBR mostly outside the DMN regions (Fig. 3b,c). The solid light-blue color mask overlaid on Fig. 3a,d (as well as on S3a and S3d) illustrates the regions where the magnitude of the NBR for the attended stimulus was significantly higher (more negative) than for the unattended stimulus (z > 2.3, after cluster-wise multiple comparisons correction). Almost all regions with NBR showed a significantly higher deactivation for attended stimuli versus unattended stimuli. While this is clearly the case for visual stimulation, it covers only a subset of the regions with deactivations during audio stimulation. This observation may be due to loud scanner noise, which can interfere with stimulation of the auditory cortex. Together, the findings suggest that attention significantly modulates the NBR in the DMN.

**Figure 4:**
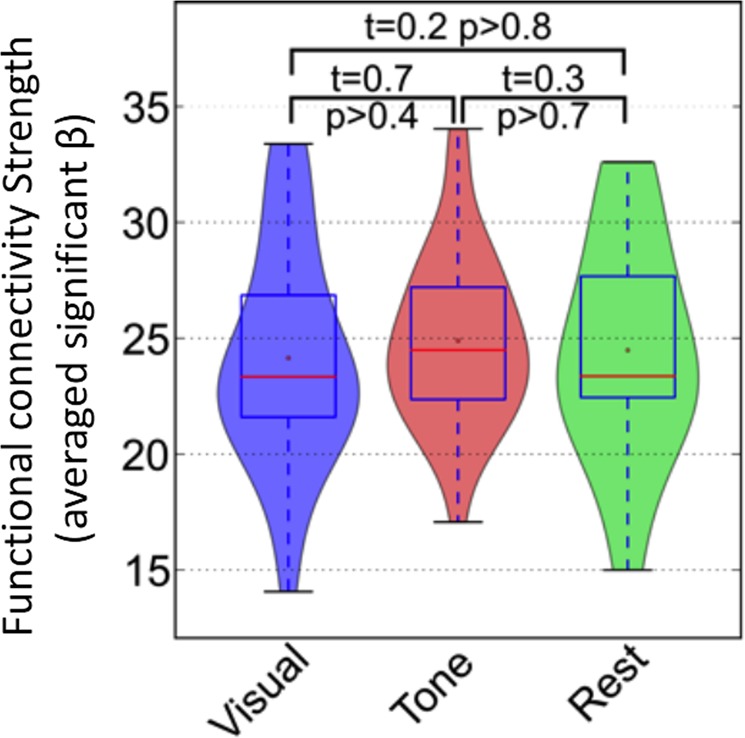
Consistency in the strength of the DMN functional connectivity during tasks and rest. The distribution of the subject-wise strength of the functional connectivity in the DMN regions extracted from attended visual (in blue), and attended audio (in red) tb-fMRI scans as well as from the rs-fMRI scan (in green) are illustrated with different violin plots. Pair-wise student t-test reveals no significant difference between any pairs of the three distributions (visual versus rest: t = 0.2, p > 0.8; audio versus rest: t = 0.3, p > 0.7; visual versus audio: t = 0.7, p > 0.4), highlighting that functional connectivity strength remains intact during tasks and rest.

Next, we assessed whether switching attention from one sensory modality to another alters the expression (coherence and magnitude) of functional connectivity in the same regions where the change in NBR was detected. We did this by comparing the strength of the DMN functional connectivity during each of the tb-fMRI scan with that during the rs-fMRI scan. Figure 4 shows the distribution of the subject-wise functional connectivity expression (given by applying a dual regression approach on ICA; see Methods) extracted from both tb-fMRI runs and the rs-fMRI scan. Unlike the NBR in the DMN, switching attention had no significant effect on the expression of the functional connectivity within the DMN (visual-attended vs. rest, t = 0.2, p > 0.8; audio-attended vs. rest, t = 0.3, p > 0.7; visual-attended vs. audio-attended, t = 0.5, p > 0.4). Together, the results presented in Figs 3 and 4 provide evidence for a disassociation between the ongoing functional connectivity and NBR in the DMN, despite being extracted from largely overlapping anatomical regions, suggesting that they reflect two distinct, but overlapping, neurophysiological processes.

### Seed-based correlation coefficient analysis

While the expression of the entire DMN functional connectivity did not show any significant alteration due to switching attention from one sensory stimulus to the next, it is still possible that an increase in one or more inter-regional coherences within the DMN could be balanced out by a concomitant reduction in other inter-regional coherences. To address this concern and also to replicate the above findings using ICA, we used a simple seed-based correlational analysis to assess any significant alteration in the inter-regional coherence within the DMN. The obtained coordinates of the centroid seed in MNI space were (−1, −53, 24.7) for the posterior cingulate, (−0.3, 60, −3.3) for the medial-orbito-frontal, and (−46.7, −67.3, 31.3)/(51.7, −66.3, 28) for the right/left angular gyri. Table 2 reports the inter-regional coherence of the DMN using subject-wise mean and standard deviation of the computed *Pearson* correlation coefficient for visual- (top) and audio-attending (middle) scans as well as the correlation between the time-course of each region with the time-course of the attended and ignored tasks. The bottom part of Table 2 reports the differences between the two mean correlations listed for visual- and audio-attended scans along with their significance level using t-statistics. While no significant difference was detected in the inter-regional coherences of the visual- and audio-attended scans (t < 0.35, P > 0.73), the correlation of the regional time-courses with the visual task was significantly altered for all regions depending on whether the task was being attended or ignored (t < −2.2, P < 0.03). In other words, while the correlation of the fMRI signal from any DMN region (e.g. posterior cingulate) with the time-course of the visual task was significantly different between the two scans, it was statistically equivalent with the other regional time-courses of the DMN. While this effect was robust and significant for visual stimuli in all DMN regions, it was only marginally significant in one region (left angular gyrus: t = 1.94, P = 0.057) for audio stimuli. Furthermore, Tables 3 and 4 list the coherence among the DMN regions in resting–state scans along with the visual attended and audio-attended scans. They also report that there is no significant difference in the inter-regional coherence of the DMN whether the participants were attending to visual/audio stimuli, or were at rest. Together, these findings not only replicate our ICA-based results, but also confirm that none of the inter-regional coherence in the DMN significantly changes with manipulation of attention (attended stimuli, unattended stimuli, or rest).

**Table 2:** The DMN inter-regional coherence measured by correlation coefficient extracted from visual-attending (top) and audio-attending (middle) scans along with their differences (bottom).

	Medial Orbito Frontal	Right Angular Gyrus	Left Angular Gyrus	Visual Task Time-course	Audio Task Time-course
	**Visual Attending**				
Posterior Cingulate	0.721 ± 0.118	0.657 ± 0.129	0.664 ± 0.128	−0.083 ± 0.124	−0.023 ± 0.11
Medial Orbito Frontal		0.546 ± 0.187	0.551 ± 0.151	−0.067 ± 0.129	−0.035 ± 0.11
Right Angular Gyrus			0.678 ± 0.126	−0.083 ± 0.095	−0.008 ± 0.094
Left Angular Gyrus				−0.088 ± 0.128	0.002 ± 0.111
Visual Stimuli					0.06 ± 0.097
	**Audio Attending**				
Posterior Cingulate	0.709 ± 0.144	0.68 ± 0.117	0.653 ± 0.12	0.027 ± 0.134	−0.058 ± 0.117
Medial Orbito Frontal		0.584 ± 0.154	0.534 ± 0.169	0.009 ± 0.13	−0.074 ± 0.114
Right Angular Gyrus			0.677 ± 0.112	0.004 ± 0.114	−0.062 ± 0.155
Left Angular Gyrus				0.034 ± 0.115	−0.056 ± 0.115
Visual Stimuli					0.094 ± 0.116
	**Differences**				
Posterior Cingulate	0.012 ± 0.126 t = 0.337, P > 0.737	−0.024 ± 0.087 t = −0.742, P > 0.461	0.011 ± 0.103 t = 0.349, P > 0.728	**−0**.**11 ± 0**.**188 t = −3**.**225**, **P < 0**.**002**	0.035 ± 0.141 t = 1.157, P > 0.252
Medial Orbito Frontal		−0.038 ± 0.144 t = −0.854, P > 0.397	0.016 ± 0.142 t = 0.383, P > 0.703	**−0**.**076 ± 0**.**2 t = −2**.**223**, **P < 0**.**03**	0.039 ± 0.141 t = 1.32, P > 0.192
Right Angular Gyrus			0.001 ± 0.103 t = 0.035, P > 0.972	**−0**.**087 ± 0**.**143 t = −3**.**159**, **P < 0**.**003**	0.054 ± 0.175 t = 1.589, P > 0.118
Left Angular Gyrus				**−0**.**122 ± 0**.**153 t = −3**.**816**, **P < 0**.**0003**	**0**.**058 ± 0**.**14 t = 1**.**942**, **P < 0**.**057**
Visual Stimuli					−0.033 ± 0.144 t = −1.191, P > 0.239

In addition, correlations between the DMN’s regional time-course and the visual and audio task time-courses are also listed along with their differences.

**Table 3:** The DMN inter-regional coherence measured by correlation coefficient extracted from visual-attending (left) and resting-state (middle) scans along with their differences (right).

	Visual Attending			Resting State			Differences		
	Medial Orbito Frontal	Right Angular Gyrus	Left Angular Gyrus	Medial Orbito Frontal	Right Angular Gyrus	Left Angular Gyrus	Medial Orbito Frontal	Right Angular Gyrus	Left Angular Gyrus
Posterior Cingulate	0.721 ± 0.118	0.657 ± 0.129	0.664 ± 0.128	0.766 ± 0.083	0.713 ± 0.083	0.673 ± 0.166	−0.046 ± 0.1 t = −1.31, P < 0.197	−0.056 ± 0.106 t = −1.511, P < 0.138	−0.01 ± 0.147 t = −0.21, P < 0.835
Medial Orbito Frontal		0.546 ± 0.187	0.551 ± 0.151		0.616 ± 0.118	0.631 ± 0.145		−0.071 ± 0.153 t = −1.307, P < 0.198	−0.08 ± 0.148 t = −1.658, P < 0.105
Right Angular Gyrus			0.678 ± 0.126			0.733 ± 0.143			−0.055 ± 0.135 t = −1.291, P < 0.204

**Table 4:** The DMN inter-regional coherence measured by correlation coefficient extracted from audio-attending (left) and resting-state (middle) scans along with their differences (right).

	Audio Attending			Resting State			Differences		
	Medial Orbito Frontal	Right Angular Gyrus	Left Angular Gyrus	Medial Orbito Frontal	Right Angular Gyrus	Left Angular Gyrus	Medial Orbito Frontal	Right Angular Gyrus	Left Angular Gyrus
Posterior Cingulate	0.709 ± 0.144	0.68 ± 0.117	0.653 ± 0.12	0.766 ± 0.083	0.713 ± 0.083	0.673 ± 0.166	−0.057 ± 0.113 t = −1.393, P < 0.171	−0.032 ± 0.1 t = −0.94, P < 0.352	−0.021 ± 0.143 t = −0.474, P < 0.638
Medial Orbito Frontal		0.584 ± 0.154	0.534 ± 0.169		0.616 ± 0.118	0.631 ± 0.145		−0.032 ± 0.136 t = −0.7, P < 0.488	−0.096 ± 0.157 t = −1.841, P < 0.073
Right Angular Gyrus			0.677 ± 0.112			0.733 ± 0.143			−0.056 ± 0.128 t = −1.407, P < 0.167

### Unlike functional connectivity, NBR in the DMN correlates with task performance

**Figure 5:**
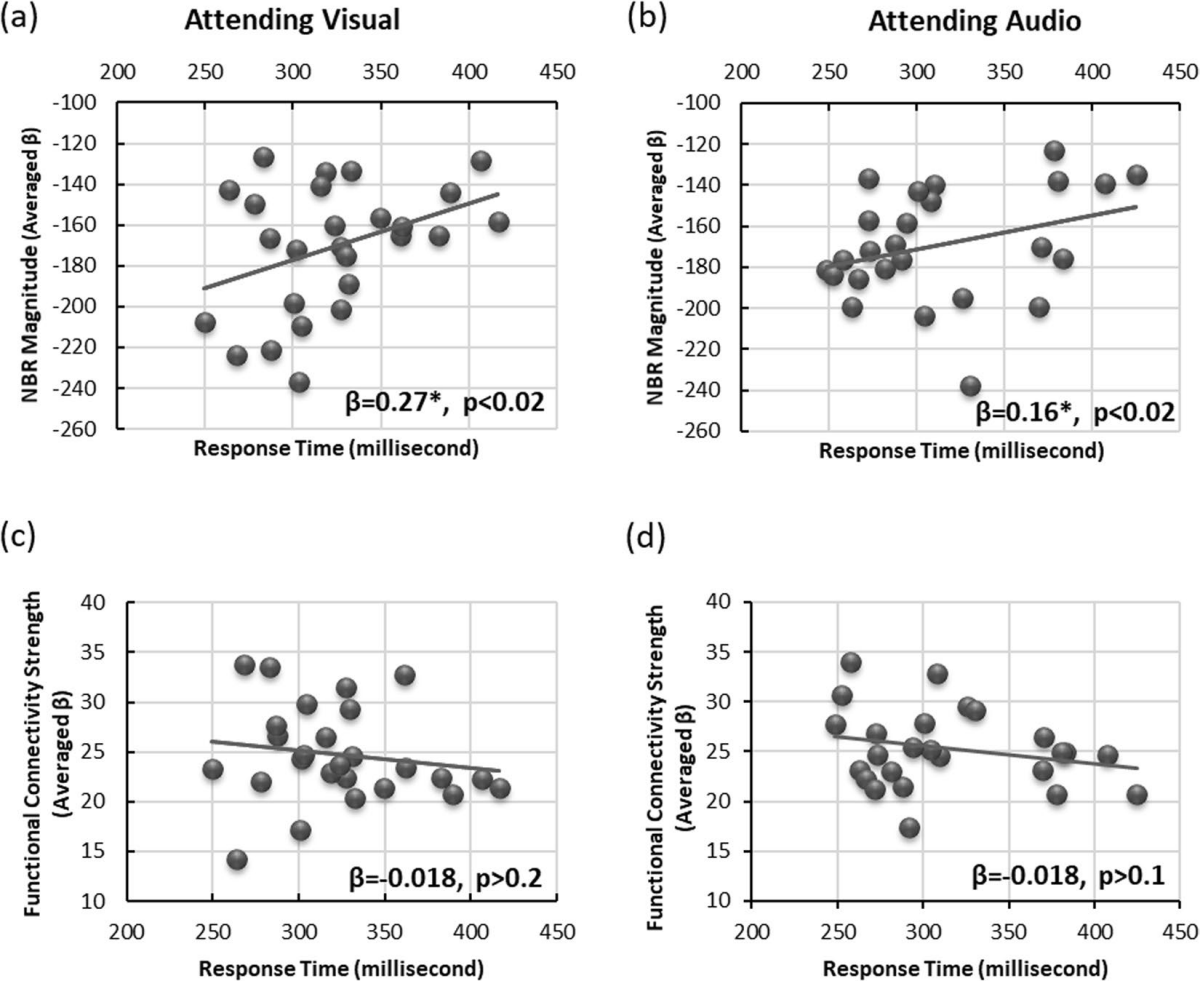
The DMN functional connectivity and NBR are differentially related to task performance. Subject-wise median response time correlates with the subject-wise magnitude of the NBR in the DMN during (**a**) attended visual (β = 0.24, p < 0.02) and (**b**) attended audio (β = 0.17, p < 0.02) tb-fMRI scans. However, it does not correlate with the subject-wise expression of functional connectivity in DMN regions during (**c**) attended visual (β = −0.018, p > 0.2) and (**d**) attended audio (β = −0.018, p > 0.01) tb-fMRI scans, providing evidence for differential level of involvement of the two fMRI measurements in task execution. Each dot represents a single subject and the line presents the linear fit to the data.

The dissociation between functional connectivity and the NBR within DMN regions raises further questions: what is the purpose of having two overlapping neurophysiological processes and is there any difference in their roles for executing a task? To examine this, we assessed the relationship between the task performance (response time) of each subject with the magnitude of its NBR, as well as the strength of its functional connectivity obtained during the two tb-fMRI scans. Figure 5 illustrates the magnitude of the NBR in the DMN regions as a function of the subjects’ median response time for visual-attended (5a) and audio-attended (5b) tb-fMRI scans. It also depicts the functional connectivity in the DMN as a function of the median response time during the same tb-fMRI scans (5c and 5d). Figure 5a,b indicates that for every 10%/7% increase in the magnitude of the NBR (greater deactivation), subjects responded 100 ms faster to the visual/audio stimulus (β = 0.24, p < 0.02 / β = 0.17, p < 0.02). In contrast, we did not find any relationship between task performance and the strength of functional connectivity in the DMN (Fig. 5c,d) extracted from the same two tb-fMRI scans (visual-attended: β = 0.018, p > 0.2; audio-attended: β = 0.017, p > 0.1). These results suggest that the neurophysiological processes represented by the NBR in the DMN might be more relevant for task execution, whereas the processes represented by ongoing functional connectivity might have more basic or auxiliary roles in the functional organization of the brain.

### Replication study using Human Connectome dataset

We replicated our findings in a different set of participants, experimental tasks, pulse sequences, and scanners. Using the publicly available Human Connectome Project dataset, often referred to as: *“unrelated-100”*^45^ and already preprocessed fMRI scans^54^, we first obtained the spatial extent and magnitude of the NBR using standard GLM analysis but with the same parameters used for processing Dataset I. We then computed group ICA on the tb-fMRI data collected during an N-back working memory task. This analysis resulted in 47 ICs, the 20th of which corresponded to activity in the DMN and accounted for 2.28% of explained variance. Running group ICA on the truncated rs-fMRI scan resulted in 42 ICs; the third IC represented the DMN and accounted for 2.87% of explained variance.

**Figure 6:**
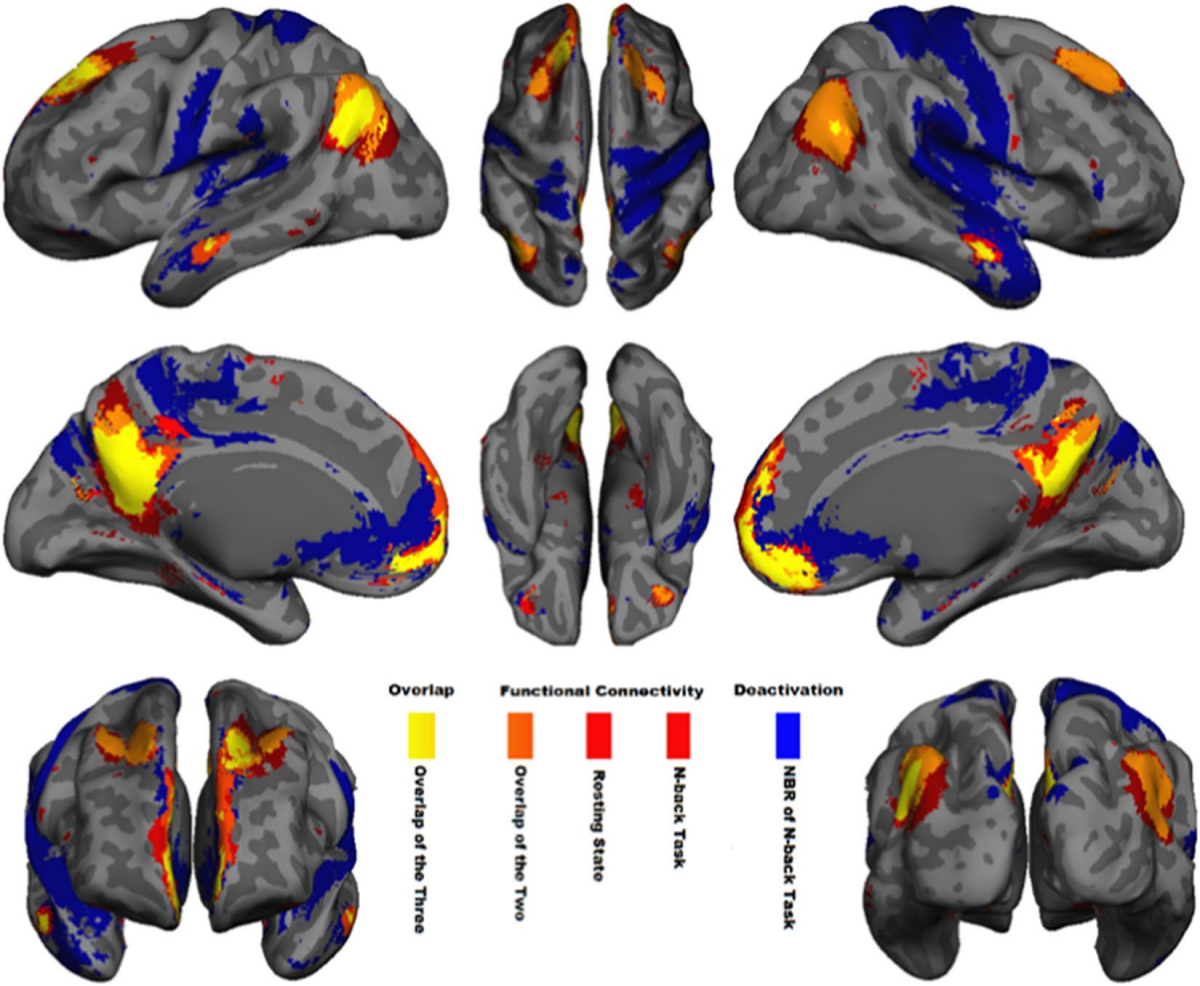
Spatial overlap between the DMN’s functional connectivity and NBR during 2-back task. Voxel-wise significant z statistics are mapped onto a semi-inflated cortical surface for a better visualization (slice-based mapping can be found in Fig. S7). Blue denotes the spatial extent of the regions with significant (z < −4 after multiple comparisons correction) NBR during 2-back working memory task. Red depicts the spatial extent of the regions with significant functional connectivity (z > 4 after multiple comparisons correction) extracted from the same 2-back tb-fMRI scan. Light red indicates the spatial extent of the regions with significant functional connectivity (z > 4 after multiple comparisons correction) extracted from the rs-fMRI scan. Orange highlights the overlap of the two functional connectivity networks. Yellow highlights the overlap of the two functional connectivity networks and the NBR during the 2-back working memory task.

Figure 6 illustrates the extent of the NBR in the 2-back tb-fMRI scan in blue (z < -4 with cluster-wise multiple comparisons correction), as well as the extent of the DMN functional connectivity extracted from the same scans in red (z > 4 with cluster-wise multiple comparisons correction). Figure 6 also illustrates the extent of the DMN functional connectivity obtained from rs-fMRI scans in red. Different overlaps of these three networks have been color-coded from orange to yellow to represent the amount of similarity among them. The slice-based version of Fig. 6 is also given in Fig. S7, where the overlap of the three masks (two functional connectivity and one NBR) is noteworthy in small bilateral hippocampal regions (pointed out by two green arrows). The extent of the NBR for the 2-back task reached far beyond the typical DMN regions. For instance, the significant NBR in the superior temporal gyrus, central sulcus, inferior frontal gyrus and posterior portion of the insula are generally not considered to be part of the DMN^4^. Since our focus is only on NBR in the DMN regions, and for consistency with the analyses of Dataset I, we excluded these non-DMN regions from this analysis.

Table 5 quantifies the pairwise spatial similarities among task-evoked NBR, task-based functional connectivity, and resting-state functional connectivity networks using the DSC, which indicated a significantly high spatial similarity between the functional connectivity networks extracted from tb-fMRI and rs-fMRI (DSC = 0.7, p < 10^−10^). Table 5 also shows a significant spatial similarity between the NBR and the functional connectivity network extracted from the same scan (DSC = 0.42, p < 10^−8^), and between the NBR and functional connectivity obtained from the rs-fMRI scan (DSC = 0.48, p < 10^−8^). Together, Fig. 6 and Table 5 provide evidence for the existence of anatomically overlapping neurophysiological processes in the DMN that are both active during a working memory task performance.

**Table 5:** Quantification of the pairwise spatial overlap of the regions with significant NBR and functional connectivity in the DMN during 2-back working memory task using Dice similarity coefficient. These regions are depicted in Fig. 6.

		Functional connectivity of DMN		NBR in DMN
		2-back Task	Resting State	2-Back Task
Functional connectivity of DMN	2-Back Task		0.697	0.408
	Resting State	0.697		0.482
NBR in DMN	2-Back Task	0.408	0.482	

**Figure 7:**
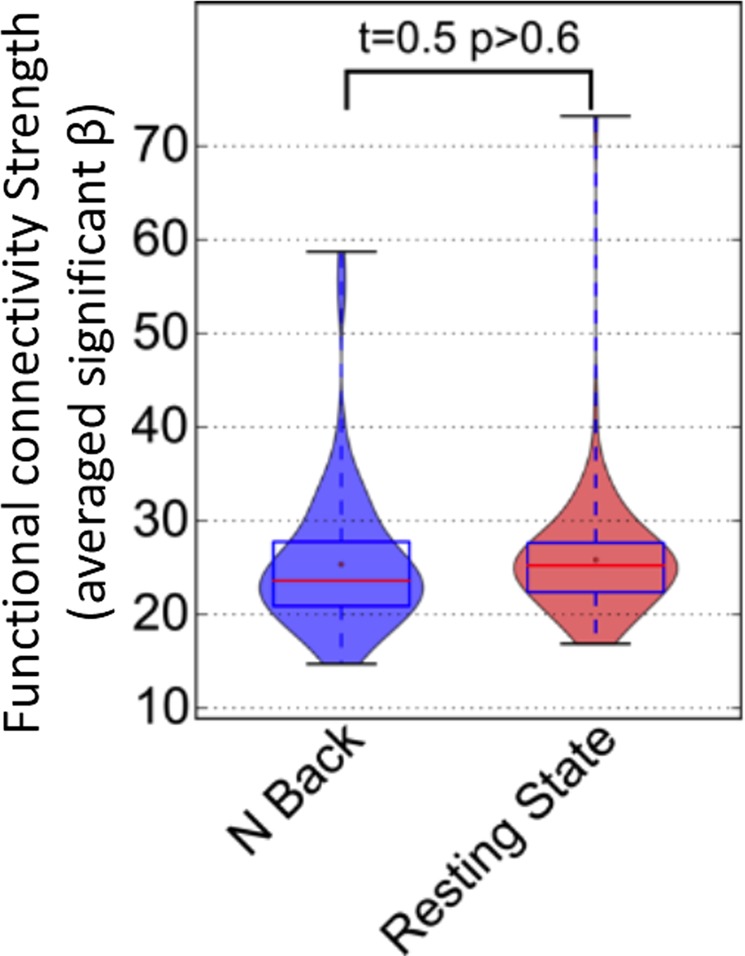
Consistency in the strength of the DMN functional connectivity during 2-back working memory task and rest. The distribution of the subject-wise strength of the functional connectivity in the DMN regions extracted from the 2-back working memory task (in blue), and from the rs-fMRI scan (in red) are illustrated with different violin plots. Pair-wise student t-test reveals no significant difference between the two distributions (t = 0.5, p > 0.6), highlighting that in comparison to rest, functional connectivity expression remains intact during working memory task.

Next, we investigated the effect of task performance on the neurophysiological process that gives rise to functional connectivity in the DMN. We examined this by comparing the expression of functional connectivity during the working memory task with the expression of the functional connectivity in the resting brain. Figure 7 shows the distribution of the strength of the functional connectivity extracted from the two scans using violin plots. As seen in Fig. 7, there is no significant difference in the subject-wise strength of DMN functional connectivity during rest and during task performance (t = 0.5, p > 0.6), replicating our finding from Dataset I. Together, the findings provide further evidence that the DMN functional connectivity represents a distinct and ongoing neurophysiological process whose coherence and magnitude are not altered by task-performance.

**Figure 8:**
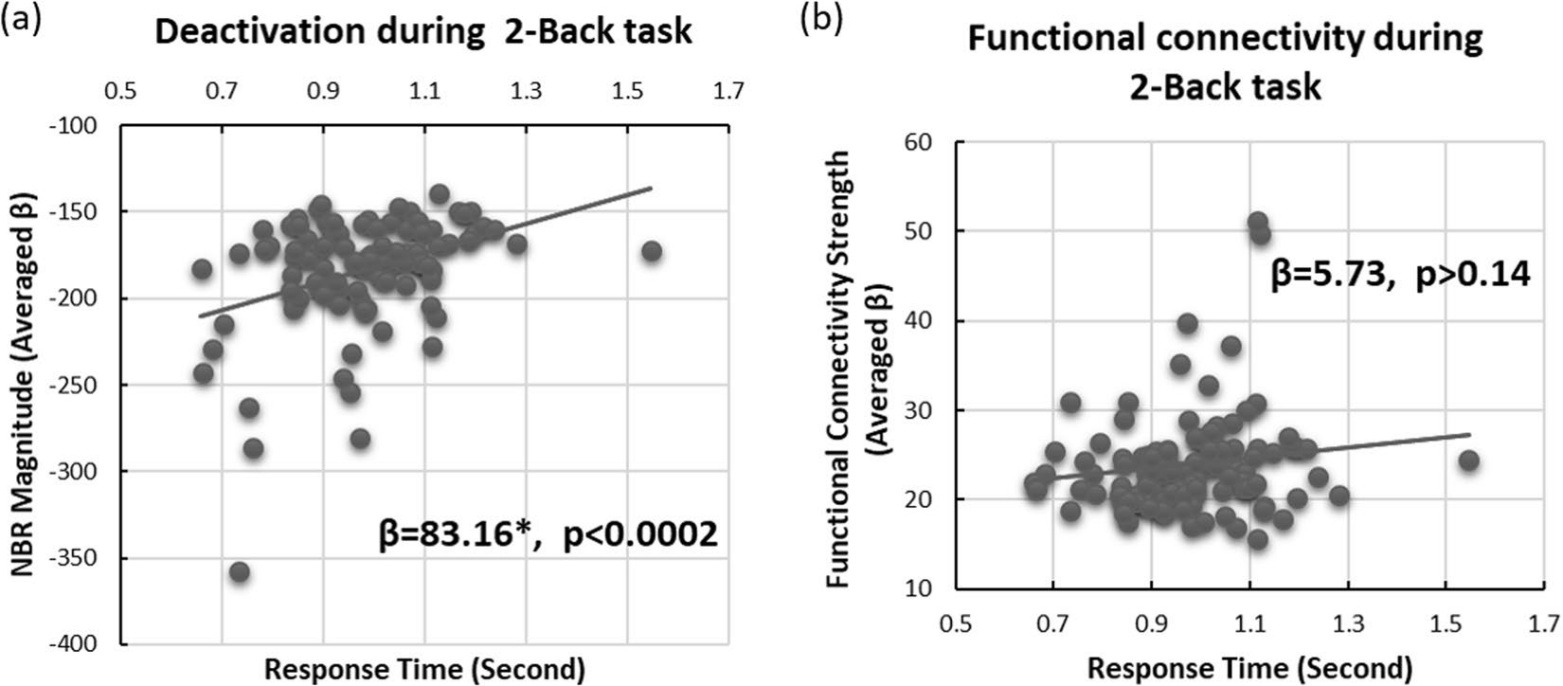
Unlike the DMN’s functional connectivity, its negative BOLD response correlates with performance during a 2-back working memory task. Subject-wise median response time correlates with the subject-wise magnitude of the NBR in the DMN regions during a 2-back working memory task (β = 83.16, p < 0.0002). However, it does not correlate with the subject-wise expression of functional connectivity in DMN regions during the same tb-fMRI scan (β = 5.73, p > 0.14), providing evidence for differential level of involvement of the two fMRI measurements in task execution. Each dot represents a single subject and the line presents the linear fit to the data.

Finally, we assessed which of the spatially overlapping and simultaneously active neurophysiological processes are more closely related to the execution of the 2-back working memory task. We examined this by investigating the relationship between task performance (response time) and the two fMRI measurements from the DMN. Similar to Dataset 1, we associated the median response time during the 2-back memory task with the magnitude of the NBR as well as the expression of functional connectivity in the DMN regions obtained from the tb-fMRI scan. Figure 8 depicts the magnitude of the NBR (8a) as well as the strength of functional connectivity (8b) as a function of the subjects’ median response time during the 2-back task. As shown in Fig. 8a, the magnitude of the NBR in DMN regions was significantly correlated with response time in the 2-back memory task, such that for every 8.3% increase in the magnitude of the NBR (more deactivation) participants responded 100 ms faster (β = 83.16, p < 0.0002). Such a relationship did not exist for functional connectivity within the same regions (β = 5.73, p > 0.14). These results replicate findings from Dataset I and support the hypothesis that NBR and functional connectivity reflect dissociable neurophysiological processes that may have different roles in the functional architecture of macro-scale networks of the human brain. Furthermore, they provide preliminary evidence that the NBR is a representative of neurophysiological processes that are more directly relevant to task performance, and that the spatially overlapping ongoing functional connectivity perhaps has an auxiliary role that may provide infrastructure for task-involved neurophysiological processes, again suggesting hierarchical functional organization in this brain network.

## Discussion

We first showed that the regions of the DMN have significant task-evoked NBR, and at the same time are functionally connected during tb-fMRI as well as rs-fMRI scans. A high degree of spatial overlap between these two fMRI measurements was notable even in the small hippocampal regions (see Fig. S7). Despite such significant spatial overlap, we also demonstrated a disassociation between the task-evoked NBR and functional connectivity, both extracted from the same DMN regions. Presentation of an unattended sensory stimulus did not induce NBR in DMN regions, whereas the identical stimulus, when it was the focus of attention, did induce strong NBR in the DMN. In contrast, whether the participants were attending/ignoring the stimulus or they were at rest had no significant effect on the functional connectivity of the DMN regions. While there are reports relating the magnitude of NBR in the DMN to task difficulty^30,57^, to the best of our knowledge, the absence of NBR in the DMN in response to unattended stimuli, which provides evidence for attention specificity of the NBR in DMN regions, has not been previously reported. The results suggest that attention to or engagement with the task should be considered a prerequisite in studies investigating task-evoked NBR in the DMN. This is particularly important for animal fMRI studies investigating the NBR, which are usually carried out under anesthesia^58^. It also highlights the importance of including the NBR in fMRI studies investigating brain-behavior relationships, rather than masking or discarding deactivations, as is often done in the literature.

To demonstrate the disassociation between functional connectivity and NBR in the DMN, we showed that the expression and extent of functional connectivity in the DMN at rest is statistically equivalent to functional connectivity in the DMN during task performance, either when attending to a visual/auditory stimulus or when performing the N-back working memory task. Despite a significant number of studies investigating the effect of task performance on functional connectivity networks, the effect of a task on functional connectivity is not completely understood in the field^43,59–61^. We recently reported that, unlike the positive BOLD response, task-evoked NBR has no effect on the functional connectivity of the DMN^44^. Here, we have complemented our previous finding by showing that switching attention from one sensory modality to another modulated the NBR in the DMN regions, while it did not have any significant effect on functional connectivity of the same regions, providing evidence for disassociation between these two fMRI measurements. To our knowledge, this disassociation has not been reported previously. Together, these results suggest that there are distinct but overlapping neurophysiological processes in the DMN. Thus, these processes may have different roles in the functionality of the DMN, and should be differentiated in studies investigating normal operation or malfunctioning of the DMN in healthy and diseased populations.

Finally, we have provided evidence that the neurophysiological processes that give rise to functional connectivity in the DMN are less relevant to task performance than the ones triggering task-evoked NBR in the same regions. We have done this by showing that the magnitude of the NBR in the DMN regions was significantly correlated with performance while the expression of functional connectivity within the DMN was not. This differential relationship with task performance not only provides further evidence that functional connectivity and task-evoked NBR reflect neurophysiological processes that have different responsibilities in the functional organization of the DMN, but also highlights the possibility that the DMN may have a multi-level or hierarchical functional architecture, such that task-related processes are dependent on the functional connectivity network (as a lower level process) to execute their duties, and the lower level neurophysiological processes would not be directly involved in task performance. However, to assess this hypothesis, further experiments are required to demonstrate that any disruption in the functional connectivity network would be propagated to the higher level processes, and would in turn cause alteration in the task-evoked NBR.

### DMN functional connectivity and task performance

While there are a few studies that relate task performance inside the fMRI scanner to the NBR in DMN regions^30,57,62^, studies relating functional connectivity to task performance inside the scanner are rare. One such study by Hampson and colleagues, using a 3-back working memory task, reported a relationship between DMN functional connectivity and task performance^63^. We were not able to replicate this result in the Connectome dataset with a 2-back memory task. However, Hampson *et al*. computed functional connectivity from tb-fMRI data using correlation analysis without regressing out task-related variability, which might have created a confound with NBR in the same regions, especially given the rather high cut-off frequency (0.2 Hz) for their low-pass filter. Another possibility that might explain the discrepancy is the difference in the performance measures. We were unable to use accuracy as our performance measure due to ceiling effects (more that 50% of the participants had accuracy above 95%) and instead used response time, whereas Hampson *et al*. were able to use accuracy since the 3-back task is more challenging. Therefore, it is also possible that our sensory-motor and 2-back working memory tasks are not challenging enough to cause any alterations in the functional connectivity network, thus a relationship between connectivity and task performance could not be detected. While further examination seems to be warranted for assessing this possibility, we need to emphasize that even the less challenging tasks did modulate the NBR in the DMN regions, which again highlights the difference between these two fMRI measurements arising from the same regions.

### DMN functional connectivity and cognition

Several studies have reported a significant relationship between the coherence of DMN functional connectivity and neuropsychological test scores administered outside the fMRI scanner^64–66^. These findings may initially seem in conflict with our proposal that functional connectivity networks are not directly involved in task performance. However, our results do not exclude a potential indirect influence of functional connectivity on task performance, particularly in regards to demanding cognitive operations. One can view functional connectivity networks as the infrastructure for the task-related networks; obviously, significant disruption in the integrity of such infrastructure will affect the function of any network that relies on such infrastructure.

Our results may also seem at odds with studies reporting disruptions in DMN functional connectivity in clinical populations, including Alzheimer’s disease^67,68^, Parkinson’s disease^69,70^, schizophrenia^71,72^, autism^73^, depression^74,75^, attention deficit and hyperactivity disorder^76,77^, and multiple sclerosis^78^. These studies provide unequivocal evidence that disruption of DMN functional connectivity is associated with disease status or level of cognition. This is also not contrary to our findings—in fact, based on our hypothesis, disruption in the functional connectivity network should cause alteration in the task-related neurophysiological processes that are directly involved in task performance, thus deterioration of cognitive ability would be warranted. However, the disassociation introduced in this work suggests that clinical populations that do not present any disruption in DMN functional connectivity may show alteration in NBR from the same regions. This raises the possibility of introducing new brain bio-markers in clinical populations.

### Methods of network extraction

Because we used two different methods to extract functional connectivity and task-based co-deactivation networks, it is reasonable to ask whether the observed disassociation can be explained by the difference in the methods used in this work. The reason we used ICA to extract functional connectivity networks is because it is challenging to extract functional connectivity networks from tb-fMRI data using the conventional correlation-based techniques. To be able to use conventional techniques, studies often discard the task period or sometime rest period from the tb-fMRI scans^79^, or remove the task-related variability by regressing out the linearly predicted BOLD response from the tb-fMRI data^59,60,80^. While none of these methods are optimal in removing the task-related variability from tb-fMRI data, ICA by definition is a blind source deconvolution technique that is considered an optimal method for separating signals from different sources^53,81^. In addition, ICA decomposition generates the voxel-wise weighting factors to compute the time-course of the fluctuations in each IC, which can subsequently be used in a dual-regression technique as predictors in a simple GLM analysis to compute not only the coherence but also the magnitude of the relationship with the IC time-course in each voxel. The point estimates obtained from the dual-regression methods are, therefore, directly comparable with the point estimates of the NBR, whereas seed-based correlations would be missing magnitude information. Nonetheless, we have computed the seed-based correlations between the three main regions of the DMN and demonstrated that while the inter-regional correlation coefficient remained unchanged across all three scans, the correlation of the signal in each region with the task was significantly altered depending on the task being attended or ignored, essentially replicating our findings using the ICA technique. Furthermore, we did regress out the task-related variability from the tb-fMRI data in our recently published study with the same sensory-motor task and showed that the functional connectivity of the DMN remains intact during task performance^44^. Therefore, it is unlikely that our findings are due to the utilization of different methods for extracting NBR and functional connectivity from DMN regions.

### Effect of attention on PBR

Figure 3 qualitatively shows that the magnitude of the positive BOLD response in the primary sensory cortices is higher for attended stimuli. This effect is more prominent in the primary auditory cortex, but is also present in the visual cortex. One might speculate that the increase in positive BOLD response for attended stimuli is the cause of the higher level of NBR in DMN regions. In other words, to have higher activation in visual/auditory cortices, the brain might be biomechanically required to further suppress activity within the DMN. To rule out this possibility, we tested the relationship between positive and negative BOLD responses for all visual/auditory stimulations while attending/unattending to the stimuli and found no significant relationship between the magnitude of the positive and negative BOLD responses. The results of this examination are summarized in Supplementary Fig. S8.

### Combining stimuli with different modalities into a single scan

Ideally, four fMRI scans were required to examine the four conditions in our fMRI experiment (visual/audio, and attended/unattended). However, we have combine attended visual stimulation with unattended audio stimulation into one scan and the inverse combination into another scan in our event-related fMRI experimental design. This design not only reduced the cost of scanning to half, but it also helped the participant to easily ignore the unattended stimulus. As a result, we were not able to explicitly test a possibility that whether the influence of an attended stimulation could be attenuated by the effect of an unattended stimulation with different modality. While further examination is required to completely role out this possibility, we argue that the likelihood of such possibility is extremely low. First, the epochs of the two combine stimulations in each scan are not temporally synchronized and they occur randomly throughout the scan. One might expect that if an effect of one condition is going to be cancelled out by another, they, at least, required to be happening at the same time. Second, we have found no evidence, in our experiments or others, that unattended sensory stimuli to cause PBR (an opposite effect) in the DMN regions, see Fig. S2. Finally, we have reported in our recently published work that removing task-related variability (attended and/or unattended) from fMRI data have no significant effect on the temporal and spatial patterns of the DMN functional connectivity hinting that the effect of any sensory stimulation on the DMN functional connectivity is negligible independent on whether it was an attended or an unattended stimulation.

### Mismatch in sample size

Only half of the subjects in Dataset I had been scanned with rs-fMRI. This is mainly because we decided to add a secondary aim, comparison with the resting-state DMN, to the study in the middle of our data acquisition, causing a mismatch in the sample sizes for some of our analyses. However, we should emphasize that the assessment and statistical inference performed for our main hypothesis (alteration of NBR despite intact functional connectivity in DMN) had matching sample sizes, and thus only for the secondary part of our hypothesis (the similarity between task-based and resting-state functional connectivity) were we required to perform our statistical analysis with mismatched sample sizes. To address this issue, we have replicated the same results using the Connectome dataset with rather large (100 subjects) and matching sample sizes, suggesting that our original findings were likely a true effect and not an artifact of mismatched sample sizes.

### Spontaneous versus task-evoked brain activity

While there is weak consensus in the field that there are different types of neuronal activity in the brain, spontaneous and task-evoked, the relationship between these different types of activity are yet to be fully discovered. Some studies have hypothesized that neuronal activities are independent of each other^18^ whereas others suggested more complex^19–22^, non-linear^23,24^, and even causal relationship^25–27^ between them. More striking is the recent fMRI findings that demonstrate significant spatial overlap between the network of functional connectivity and task-evoked BOLD responses^40,43^. However, the temporal and spatial association or disassociation between these two types of fMRI measurements are not completely understood. In the present study, our focus was only on the DMN, and we demonstrated that despite observing a strong spatial association between task-evoked NBR and functional connectivity in the DMN, there is a robust temporal disassociation between them. Our results suggest that at least for the DMN, the functional connectivity and NBR are representative of two distinct neurophysiological processes, despite being characterized by significant spatial overlap. The method used here is not applicable for other functional connectivity networks since only the DMN is strongly associated with the NBR. Further experiments and technical developments are warranted to show the same temporal disassociation between functional connectivity and task-evoked BOLD response in the remaining macro-scale networks of the human brain.

## Conclusion

The evidence presented in this study clearly establishes a disassociation between the DMN functional connectivity and an overlapping network of regions showing task-related deactivation. The results imply that functional connectivity and NBR are reflective of two separate but overlapping neurophysiological processes taking place in the DMN. We also found that participants’ task performance was associated with the magnitude of the NBR in the DMN regions, but not with expression of the DMN functional connectivity. We speculate that these dissociable processes comprise a nested or hierarchical system in which the NBR is indicative of a higher level neurophysiological process than the one measured by functional connectivity. We conclude that NBR and functional connectivity in the DMN regions play different roles in the functional architecture of the DMN, and should thus be distinguished in studies investigating brain-behavior relationships in healthy and clinical populations.

## Supplementary information

Supplementary Materials

## References

[1] Raichle ME (2001). A default mode of brain function. Proc. Natl. Acad. Sci. USA.

[2] Shulman GL (1997). Common Blood Flow Changes across Visual Tasks: 11. Decreases in Cerebral Cortex. J Cogn Neurosci.

[3] Buckner, R. L. & Vincent, J. L. Unrest at rest: default activity and spontaneous network correlations. *Neuroimage* **37**, 1091–6; discussion 1097–9 (2007). 10.1016/j.neuroimage.2007.01.010 17368915

[4] Buckner RL, Andrews-Hanna JR, Schacter DL (2008). The brain’s default network: anatomy, function, and relevance to disease. Ann. N. Y. Acad. Sci..

[5] Anticevic A (2012). The role of default network deactivation in cognition and disease. Trends Cogn. Sci..

[6] van Eimeren, T., Monchi, O., Ballanger, B. & Strafella, A. P. Dysfunction of the Default Mode Network in Parkinson Disease. *Arch*. *Neurol*. **66** (2009). 10.1001/archneurol.2009.97 PMC2972248 19597090

[7] Spencer MD (2012). Failure to deactivate the default mode network indicates a possible endophenotype of autism. Mol. Autism.

[8] Zhou L (2016). Inefficient DMN Suppression in Schizophrenia Patients with Impaired Cognitive Function but not Patients with Preserved Cognitive Function. Sci. Rep..

[9] Sheline YI (2009). The default mode network and self-referential processes in depression. Proc. Natl. Acad. Sci..

[10] Hamel E (2004). Cholinergic modulation of the cortical microvascular bed. Prog. Brain Res..

[11] Attwell D, Iadecola C (2002). The neural basis of functional brain imaging signals. Trends Neurosci..

[12] Raichle M, Mintun M (2006). Brain work and brain imaging. Annu. Rev. Neurosci..

[13] Logothetis NK, Pauls J, Augath M, Trinath T, Oeltermann A (2001). Neurophysiological investigation of the basis of the fMRI signal. Nature.

[14] Raichle ME (1998). Behind the scenes of functional brain imaging: a historical and physiological perspective. Proc. Natl. Acad. Sci. USA.

[15] Biswal B, Yetkin FZ, Haughton VM, Hyde JS (1995). Functional connectivity in the motor cortex of resting human brain using echo-planar MRI. Magn. Reson. Med..

[16] Greicius MD, Krasnow B, Reiss AL, Menon V (2003). Functional connectivity in the resting brain: a network analysis of the default mode hypothesis. Proc. Natl. Acad. Sci. USA.

[17] van den Heuvel MP, Hulshoff Pol HE (2010). Exploring the brain network: a review on resting-state fMRI functional connectivity. Eur. Neuropsychopharmacol..

[18] Fox MD, Snyder AZ, Zacks JM, Raichle ME (2006). Coherent spontaneous activity accounts for trial-to-trial variability in human evoked brain responses. Nat. Neurosci..

[19] Nierhaus T, Schön T, Becker R, Ritter P, Villringer A (2009). Background and evoked activity and their interaction in the human brain. Magn. Reson. Imaging.

[20] Becker R, Reinacher M, Freyer F, Villringer A, Ritter P (2011). How Ongoing Neuronal Oscillations Account for Evoked fMRI Variability. J. Neurosci..

[21] Scheeringa R, Mazaheri A, Bojak I, Norris DG, Kleinschmidt A (2011). Modulation of Visually Evoked Cortical fMRI Responses by Phase of Ongoing Occipital Alpha Oscillations. J. Neurosci..

[22] Di X, Biswal BB (2019). Toward Task Connectomics: Examining Whole-Brain Task Modulated Connectivity in Different Task Domains. Cereb. Cortex.

[23] He BJ (2013). Spontaneous and Task-Evoked Brain Activity Negatively Interact. J. Neurosci..

[24] Mayhew SD, Ostwald D, Porcaro C, Bagshaw AP (2013). Spontaneous EEG alpha oscillation interacts with positive and negative BOLD responses in the visual–auditory cortices and default-mode network. Neuroimage.

[25] Fox MD, Snyder AZ, Vincent JL, Raichle ME (2007). Intrinsic Fluctuations within Cortical Systems Account for Intertrial Variability in Human Behavior. Neuron.

[26] Sadaghiani S, Hesselmann G, Friston KJ, Kleinschmidt A (2010). The relation of ongoing brain activity, evoked neural responses, and cognition. Front. Syst. Neurosci..

[27] Sadaghiani S (2010). Intrinsic Connectivity Networks, Alpha Oscillations, and Tonic Alertness: A Simultaneous Electroencephalography/Functional Magnetic Resonance Imaging Study. J. Neurosci..

[28] Pihlajamäki M (2010). Evidence of Altered Posteromedial Cortical fMRI Activity in Subjects at Risk for Alzheimer Disease. Alzheimer Dis. Assoc. Disord..

[29] Andrews-Hanna JR, Reidler JS, Sepulcre J, Poulin R, Buckner RL (2010). Functional-anatomic fractionation of the brain’s default network. Neuron.

[30] Greicius MD, Menon V (2004). Default-mode activity during a passive sensory task: uncoupled from deactivation but impacting activation. J. Cogn. Neurosci..

[31] Sperling RA (2009). Amyloid Deposition Is Associated with Impaired Default Network Function in Older Persons without Dementia. Neuron.

[32] Morcom AM, Fletcher PC (2007). Does the brain have a baseline? Why we should be resisting a rest. Neuroimage.

[33] Fair DA (2008). The maturing architecture of the brain’s default network. PNAS.

[34] Wise RG, Ide K, Poulin MJ, Tracey I (2004). Resting fluctuations in arterial carbon dioxide induce significant low frequency variations in BOLD signal. Neuroimage.

[35] Birn RM, Diamond JB, Smith MA, Bandettini PA (2006). Separating respiratory-variation-related fluctuations from neuronal-activity-related fluctuations in fMRI. Neuroimage.

[36] Sormaz M (2018). Default mode network can support the level of detail in experience during active task states. Proc. Natl. Acad. Sci. USA.

[37] Andrews-Hanna JR, Smallwood J, Spreng RN (2014). The default network and self-generated thought: component processes, dynamic control, and clinical relevance. Ann. N. Y. Acad. Sci..

[38] Laird AR (2011). Behavioral interpretations of intrinsic connectivity networks. J. Cogn. Neurosci..

[39] Toro R, Fox PT, Paus T (2008). Functional coactivation map of the human brain. Cereb. Cortex.

[40] Smith SM (2009). Correspondence of the brain’s functional architecture during activation and rest. Proc. Natl. Acad. Sci. USA.

[41] Crossley NA (2013). Cognitive relevance of the community structure of the human brain functional coactivation network. Proc. Natl. Acad. Sci..

[42] Di X, Gohel S, Kim EH, Biswal BB (2013). Task vs. rest-different network configurations between the coactivation and the resting-state brain networks. Front. Hum. Neurosci..

[43] Power JD (2011). Functional Network Organization of the Human Brain. Neuron.

[44] Razlighi QR (2018). Task-Evoked Negative BOLD Response in the Default Mode Network Does Not Alter Its Functional Connectivity. Front. Comput. Neurosci..

[45] Van Essen DC (2013). The WU-Minn Human Connectome Project: An overview. Neuroimage.

[46] Barch DM (2013). Function in the human connectome: Task-fMRI and individual differences in behavior. Neuroimage.

[47] Smith SM (2013). Resting-state fMRI in the Human Connectome Project. Neuroimage.

[48] Caceres A, Hall DL, Zelaya FO, Williams SCR, Mehta MA (2009). Measuring fMRI reliability with the intra-class correlation coefficient. Neuroimage.

[49] Drobyshevsky A, Baumann SB, Schneider W (2006). A rapid fMRI task battery for mapping of visual, motor, cognitive, and emotional function. Neuroimage.

[50] Desikan RS (2006). An automated labeling system for subdividing the human cerebral cortex on MRI scans into gyral based regions of interest. Neuroimage.

[51] Beckmann CF, Smith SM (2004). Probabilistic Independent Component Analysis for Functional Magnetic Resonance Imaging. IEEE Trans. Med. Imaging.

[52] T. Minka. *Automatic choice of dimensionality for PCA*. (2000).

[53] Beckmann M, Filippini, Smith (2009). Group comparison of resting-state FMRI data using multi-subject ICA and dual regression. Neuroimage.

[54] Glasser MF (2013). The minimal preprocessing pipelines for the Human Connectome Project. Neuroimage.

[55] Vann SD, Aggleton JP, Maguire EA (2009). What does the retrosplenial cortex do?. Nat. Rev. Neurosci..

[56] Cauda F (2010). Functional Connectivity of the Posteromedial Cortex. PLoS One.

[57] Hu Y, Chen X, Gu H, Yang Y (2013). Resting-State Glutamate and GABA Concentrations Predict Task-Induced Deactivation in the Default Mode Network. J. Neurosci..

[58] Shmuel A (2002). Sustained Negative BOLD, Blood Flow and Oxygen Consumption Response and Its Coupling to the Positive Response in the Human Brain. Neuron.

[59] Cole MW, Bassett DS, Power JD, Braver TS, Petersen SE (2014). Intrinsic and task-evoked network architectures of the human brain. Neuron.

[60] Gratton C, Laumann TO, Gordon EM, Adeyemo B, Petersen SE (2016). Evidence for Two Independent Factors that Modify Brain Networks to Meet Task Goals. Cell Rep..

[61] Kaufmann T (2017). Task modulations and clinical manifestations in the brain functional connectome in 1615 fMRI datasets. Neuroimage.

[62] Calhoun VD (2002). Different activation dynamics in multiple neural systems during simulated driving. Hum. Brain Mapp..

[63] Hampson M, Driesen NR, Skudlarski P, Gore JC, Constable RT (2006). Brain Connectivity Related to Working Memory Performance. J. Neurosci..

[64] Razlighi Qolamreza R., Habeck Christian, Steffener Jason, Gazes Yunglin, Zahodne Laura B., MacKay-Brandt Anna, Stern Yaakov (2013). Unilateral disruptions in the default network with aging in native space. Brain and Behavior.

[65] Seeley WW (2007). Dissociable Intrinsic Connectivity Networks for Salience Processing and Executive Control. J. Neurosci..

[66] Shaw EE, Schultz AP, Sperling RA, Hedden T (2015). Functional Connectivity in Multiple Cortical Networks Is Associated with Performance Across Cognitive Domains in Older Adults. Brain Connect..

[67] Greicius MD, Srivastava G, Reiss AL, Menon V (2004). Default-mode network activity distinguishes Alzheimer’s disease from healthy aging: evidence from functional MRI. Proc Natl Acad Sci USA.

[68] Wu X (2011). Altered default mode network connectivity in Alzheimer’s disease–a resting functional MRI and Bayesian network study. Hum. Brain Mapp..

[69] Tessitore A (2012). Default-mode network connectivity in cognitively unimpaired patients with Parkinson disease. Neurology.

[70] van Eimeren T, Monchi O, Ballanger B, Strafella AP (2009). Dysfunction of the default mode network in Parkinson disease: a functional magnetic resonance imaging study. Arch. Neurol..

[71] Ongür D (2010). Default mode network abnormalities in bipolar disorder and schizophrenia. Psychiatry Res..

[72] Venkataraman A, Whitford TJ, Westin C-F, Golland P, Kubicki M (2012). Whole brain resting state functional connectivity abnormalities in schizophrenia. Schizophr. Res..

[73] Assaf M (2010). Abnormal functional connectivity of default mode sub-networks in autism spectrum disorder patients. Neuroimage.

[74] Bluhm R (2009). Resting state default-mode network connectivity in early depression using a seed region-of-interest analysis: Decreased connectivity with caudate nucleus. Psychiatry Clin. Neurosci..

[75] Zhu X (2012). Evidence of a dissociation pattern in resting-state default mode network connectivity in first-episode, treatment-naive major depression patients. Biol. Psychiatry.

[76] Fair DA (2010). Atypical Default Network Connectivity in Youth with Attention-Deficit/Hyperactivity Disorder. Biol. Psychiatry.

[77] Uddin LQ (2008). Network homogeneity reveals decreased integrity of default-mode network in ADHD. J. Neurosci. Methods.

[78] Leavitt Victoria M., Paxton Jessica, Sumowski James F. (2014). Default Network Connectivity Is Linked to Memory Status in Multiple Sclerosis. Journal of the International Neuropsychological Society.

[79] Krienen FM, Yeo BTT, Buckner RL (2014). Reconfigurable task-dependent functional coupling modes cluster around a core functional architecture. Philos. Trans. R. Soc. Lond. B. Biol. Sci..

[80] Fair DA (2007). A method for using blocked and event-related fMRI data to study “resting state” functional connectivity. Neuroimage.

[81] Calhoun VD (2001). Spatial and temporal independent component analysis of functional MRI data containing a pair of task-related waveforms. Hum. Brain Mapp..

