# Supplementary material for: Task-evoked Negative BOLD Response and Functional Connectivity in the Default Mode Network are Representative of Two Overlapping but Separate Neurophysiological Processes
Source: Sci Rep. 2019 Oct 9;9:14473. doi: 10.1038/s41598-019-50483-8 (PMC6785640; doi:10.1038/s41598-019-50483-8)
Supplement: Supplementary file 1 — Supplementary Materials [file 41598_2019_50483_MOESM1_ESM.pdf]

Supplementary Materials

Title:

Task-evoked Negative BOLD Response and Functional Connectivity in the Default Mode Network are Representative of Two Overlapping but Separate Neurophysiological Processes

Authors:

David B. Parker[1], Qolamreza R. Razlighi[1,2,3]*

[1] Department of Biomedical Engineering, Columbia University, New York, NY 10032

[2] Department of Neurology, College of Physicians and Surgeons, Columbia University, New York, NY 10032

[3] Taub Institute for research on Alzheimer's disease and the aging brain, Columbia University, New York, NY 10032

* Corresponding Author, Quantitative Neuroimaging Laboratory, Division of Cognitive Neuroscience, Department of Neurology, Columbia University. Address: 630 West 168th St, P&S 16, New York, NY 10032, USA. Phone: +1 212-342-1352, Fax: +1 212-342-1838, Email: qr2108@columbia.edu.

Figure S1

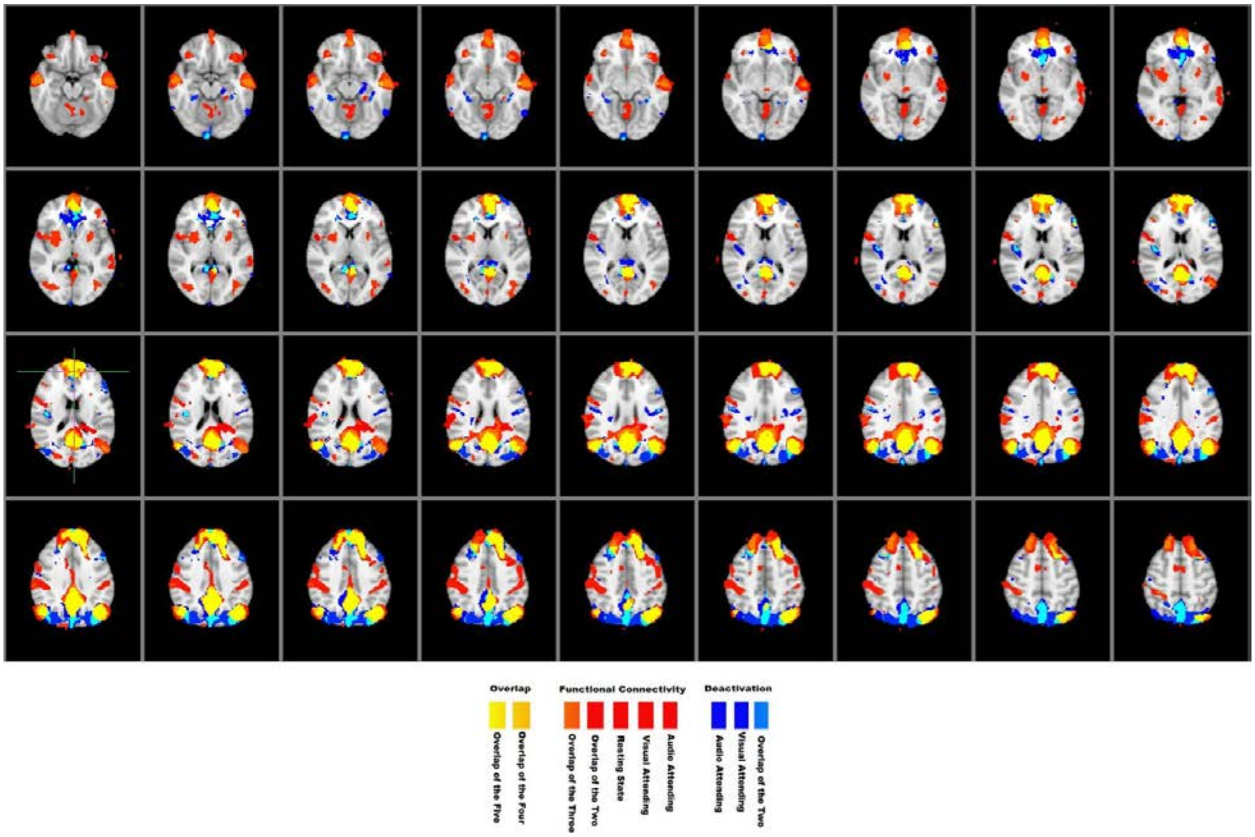

Figure S1. **Slice-based illustration of spatial overlap between regions with significant negative BOLD response as well as the functional connectivity in the DMN.** Voxel-wise significant z statistics ($|z|>4$ after multiple-comparisons correction) are mapped onto 36 axial slices of the brain as an alternative visualization for efficient illustration of the subcortical regions and buried sulci. Dark blue denotes the spatial extent of the regions with significant ($z<-4$ after multiple-comparisons correction) NBR to visual- and audio-attended stimuli during two separate tb-fMRI scans. Light blue highlights their overlap. Red indicates the spatial extent of the regions with significant functional connectivity ($z>4$ after multiple comparisons correction) extracted from two tb-fMRI scans (visual-attended and audio-attended tasks) as well as one rs-fMRI scan. Light red and orange highlight the overlap of the two and three functional connectivity networks, respectively. Dark Yellow and yellow highlight the extent of overlap of four and all five aforementioned networks, respectively.

Figure S2

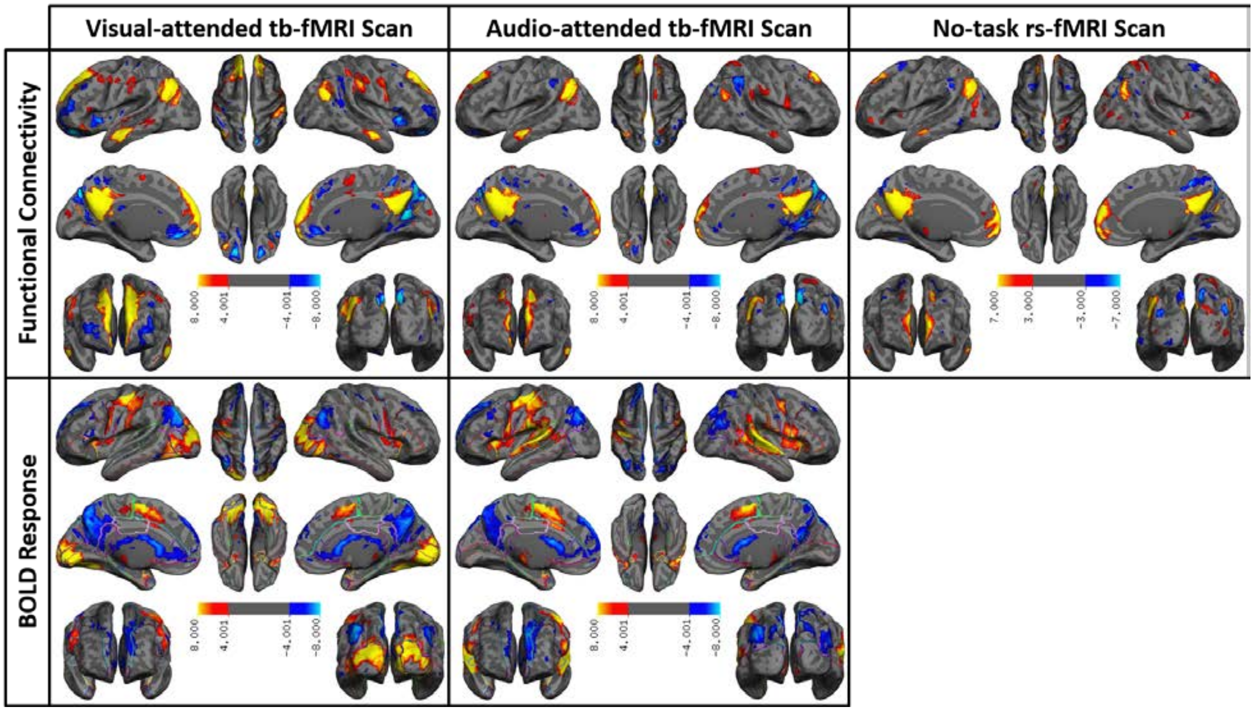

Figure S2. **Spatial pattern of regions with significant NBR and functional connectivity in DMN.** Voxel-wise significant z statistics are mapped onto a semi-inflated cortical surface individually for each functional connectivity (top raw) and NBR (bottom raw) spatial pattern obtained from the two tb-fMRI scans (left two columns) and from the rs-fMRI scan (right column).

Figure S3

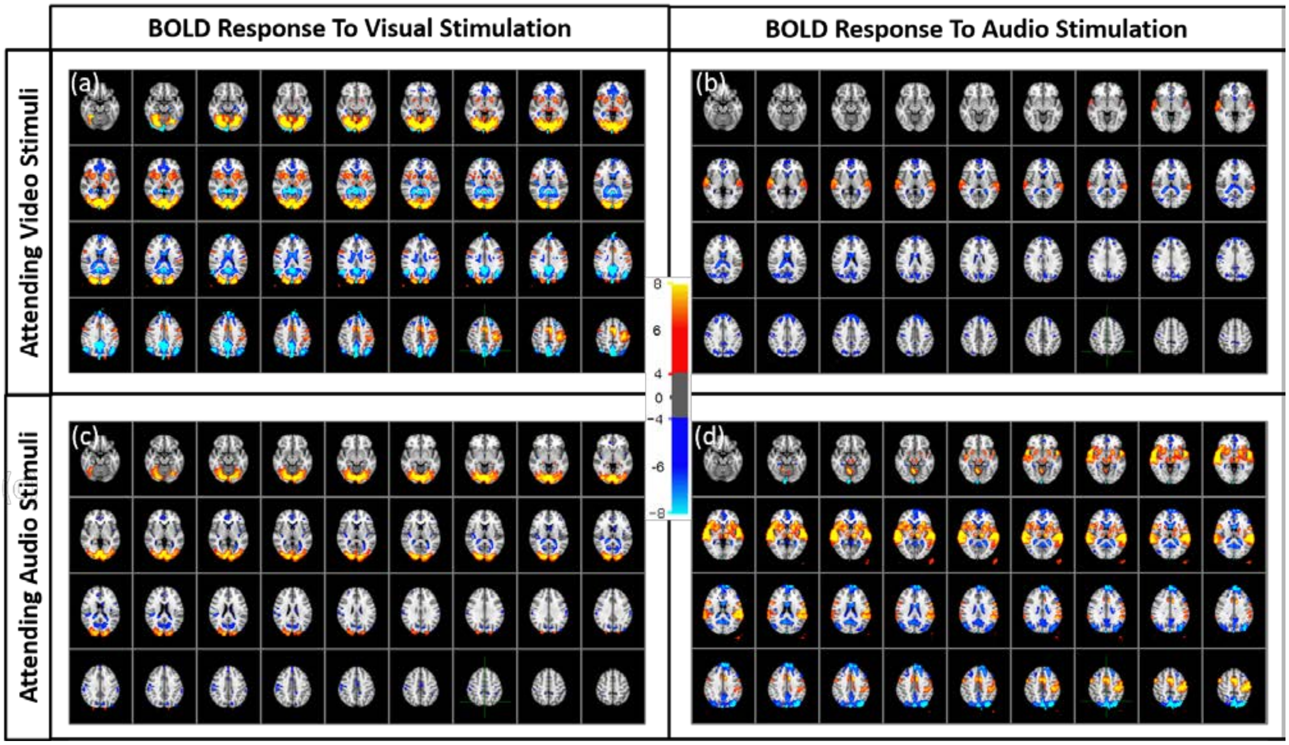

Figure S3. **Slice-based illustration of the attention-specificity of the negative BOLD response in the DMN regions**. Spatial extent of the voxels with significant PBR (activated) and NBR (deactivated) during sensory-motor tasks using z-statistics thresholded at |z|>4 for a) attended visual, b) unattended audio, c) unattended visual, and d) attended audio stimuli are mapped onto 36 axial slices of the brain. The z-statistics for PBR are color-coded with warm colors (red to yellow), and those for NBR with cold colors (blue to light blue). The solid, light blue color represents the mask of the regions that has significantly higher magnitude of the NBR for attended stimuli versus unattended stimuli (z>2.3, after cluster-wise multiple-comparisons correction). Note that almost no significant NBR (deactivation) is present for unattended stimuli.

Figure S4

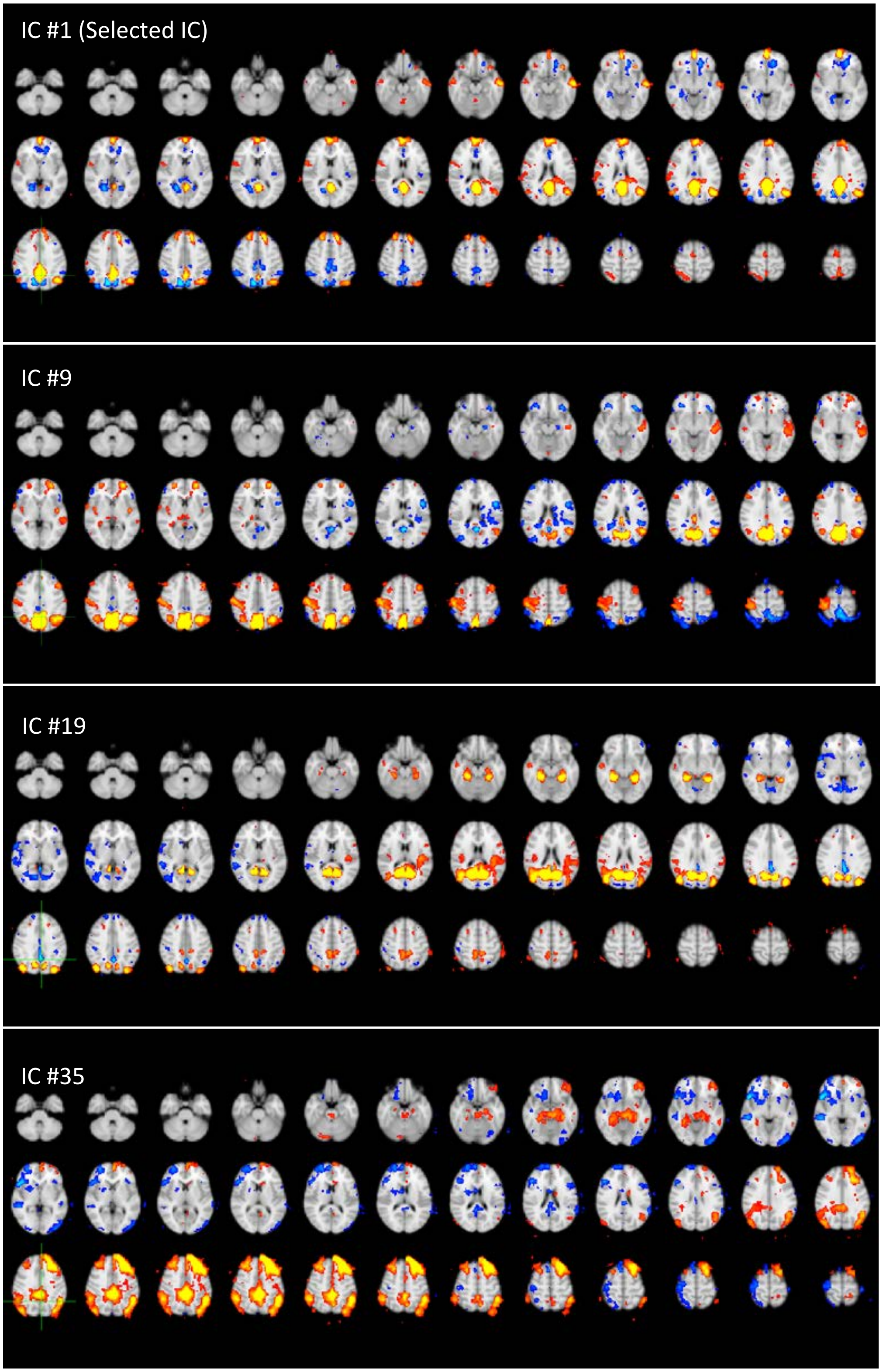

Figure S4. **The spatial pattern of all independent components extracted from video-attended scan that demonstrated more than 20% spatial overlap with the pattern of the NBR**.

Figure S5

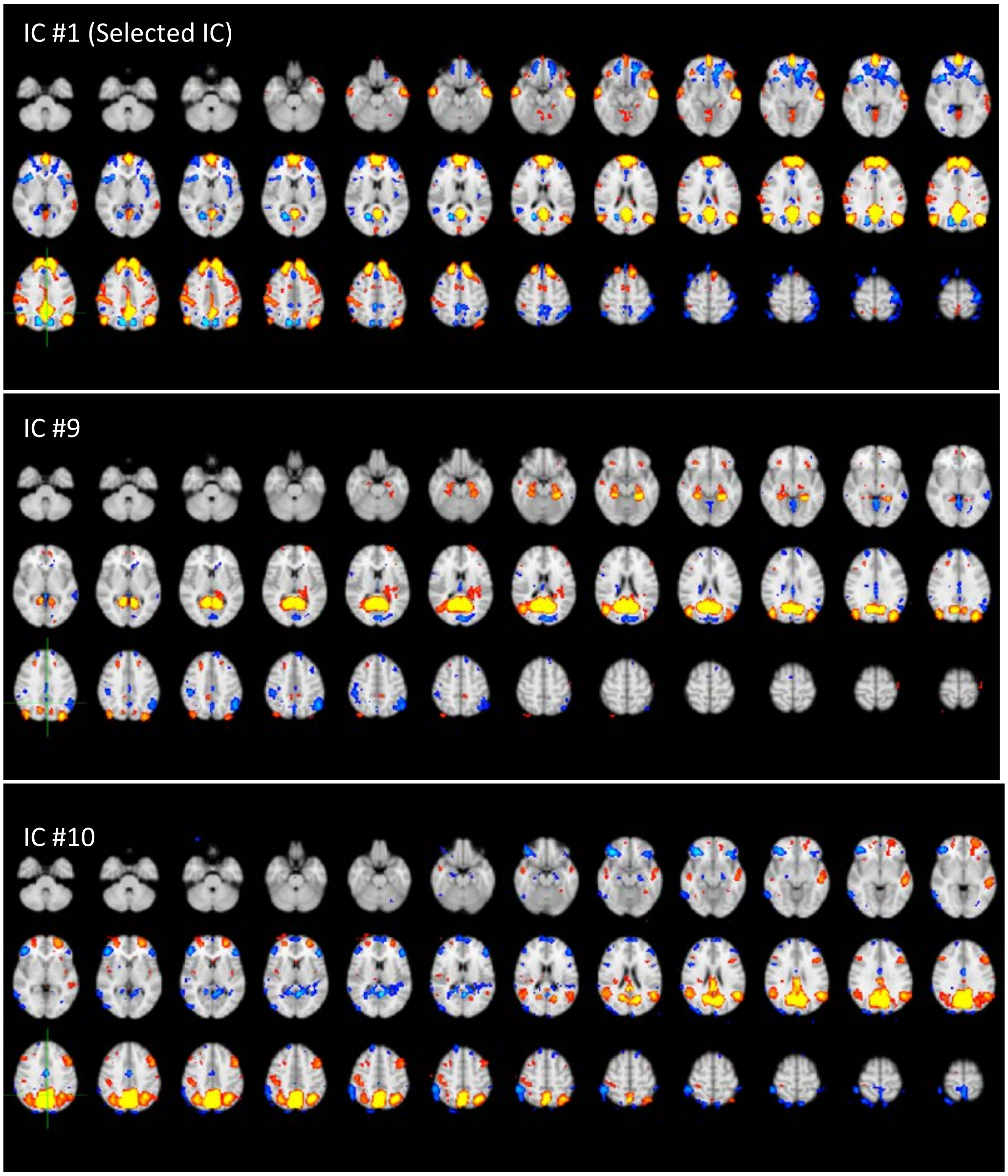

Figure S5. **The spatial pattern of all independent components extracted from the audio-attending scan that demonstrated more than 20% spatial overlap with the pattern of the NBR**.

Figure S6

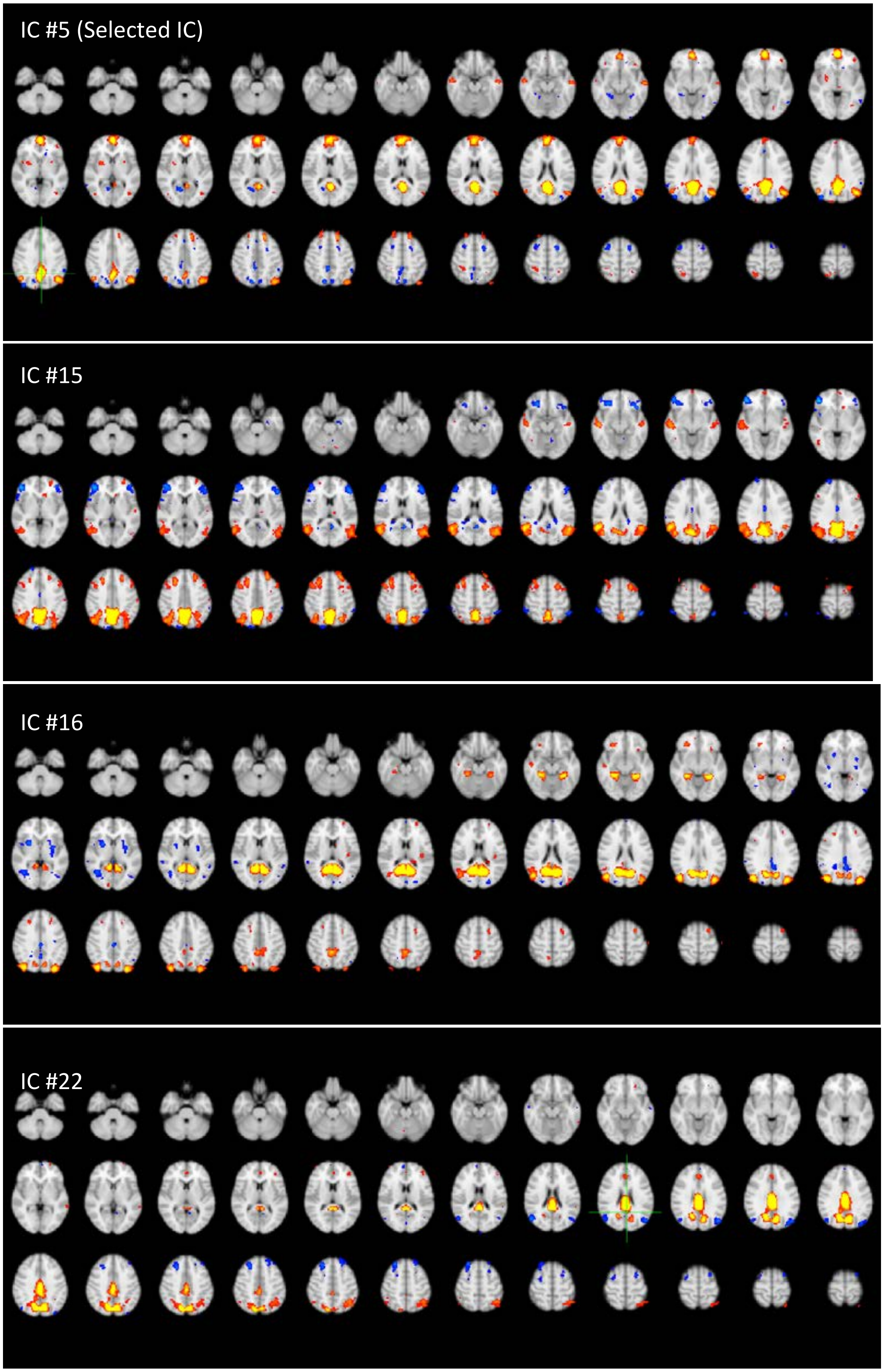

Figure S6. **The spatial pattern of all independent components extracted from the resting-state scan that demonstrated more than 20% spatial overlap with the pattern of the NBR**.

Figure S7

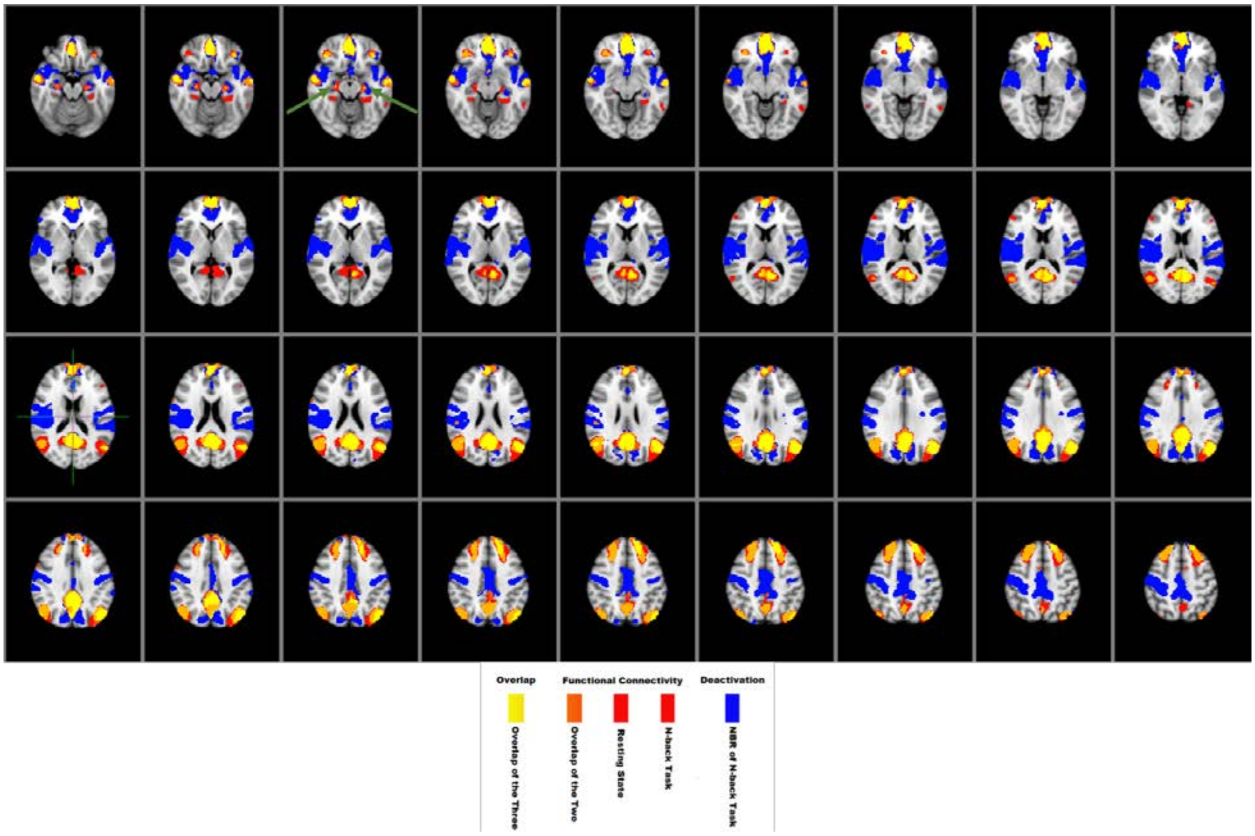

Figure S7. **Slice-based illustration of spatial overlap between the DMN's functional connectivity and NBR during 2-back task.** The voxel-wise significant z statistics are mapped onto 36 axial slices of the brain as an alternative visualization for better illustration of the subcortical regions and buried sulci. Blue denotes the spatial extent of the regions with significant NBR ($z<-4$ after multiple-comparisons correction) during 2-back working memory task. Red: indicates the spatial extent of the regions with significant functional connectivity ($z>4$ after multiple-comparisons correction) extracted from the same 2-back tb-fMRI scan. Light red denotes the spatial extent of the regions with significant functional connectivity ($z>4$ after multiple comparisons correction) extracted from the rs-fMRI scan. Orange highlights the overlap of the two functional connectivity networks. Yellow depicts the overlap of the two functional connectivity networks and the NBR during the 2-back working memory task. Green arrows pinpoint bilateral small hippocampal regions where all three mask are overlapping highlighting the high level of spatial overlap between NBR and functional connectivity in the DMN regions.

Figure S8

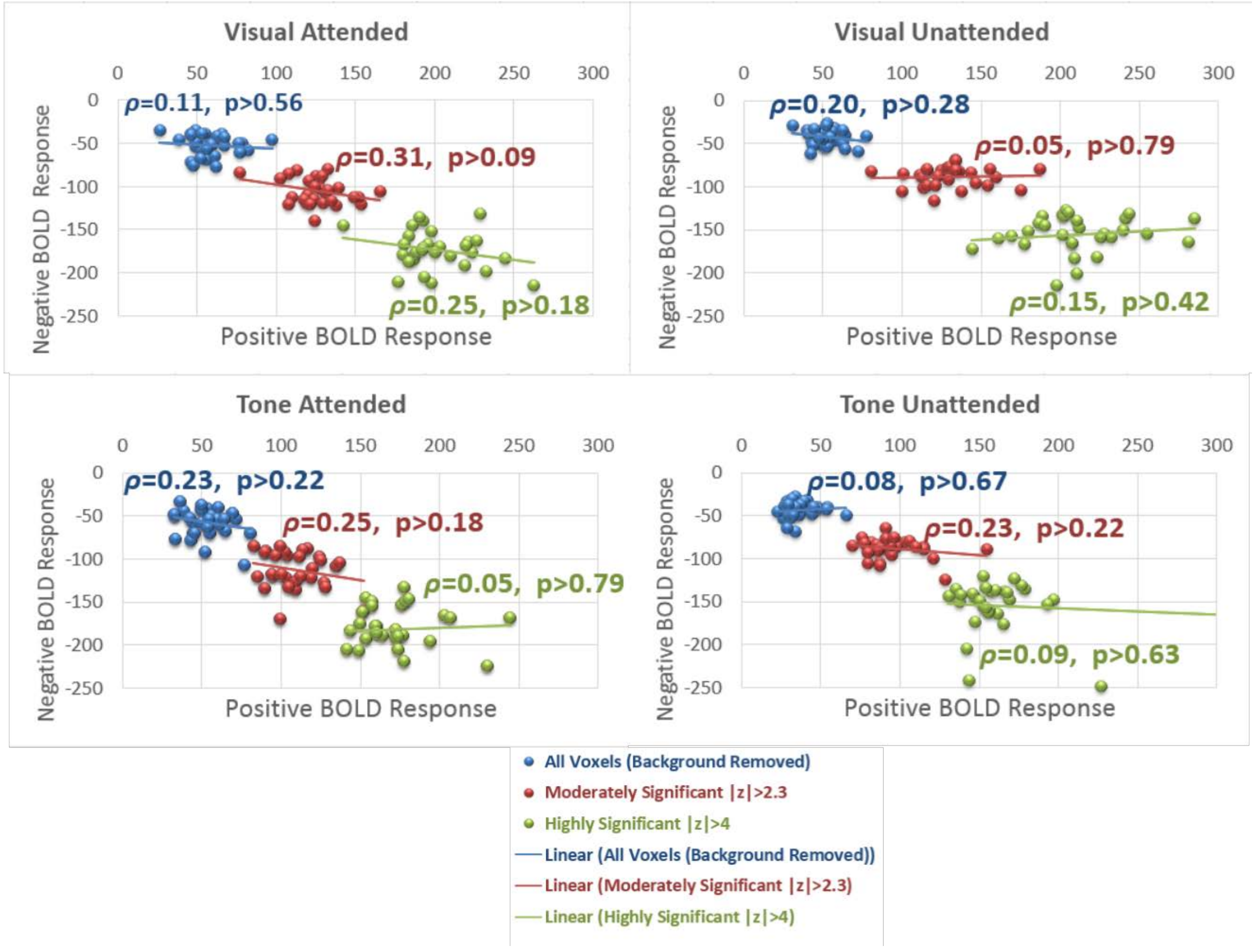

Figure S8. **The magnitude of negative BOLD response does not correlate with the magnitude of positive BOLD response.** Illustrating the relationship between the PBR and NBR for attended visual (top-left), unattended visual (top-right), attended audio (bottom-right), and unattended audio (bottom-left) stimuli. The comparisons are performed for voxels with three different levels of significance; blue: all voxels where only background noise is removed, Red: voxels with moderate level of significance $|z|>2.3$, and green: voxels with high level of significance $|z|>4$. While there is one marginally significant case for attended visual stimuli and with moderately significant voxels ($\rho=0.31$, $p>0.09$), we could not find any relationship between the magnitude of PBR (activations) and NBR (deactivations) in our dataset.
